# Vermicular Eutectic Multi‐Principal Element Alloy with Exceptional Strength and Ductility

**DOI:** 10.1002/advs.202501150

**Published:** 2025-04-03

**Authors:** Liufei Huang, Yicheng Han, Yaoning Sun, A. S. L. Subrahmanyam Pattamatta, Junhua Luan, Qing Wang, Congcong Ren, Yuanfeng Zhou, Jinfeng Li, Hengwei Luan, Peter K. Liaw, Jian Lu

**Affiliations:** ^1^ Institute of Materials China Academy of Engineering Physics Mianyang 621908 China; ^2^ School of Mechanical Engineering Xinjiang University Urumqi 830017 China; ^3^ CityU‐Shenzhen Futian Research Institute Shenzhen 518045 China; ^4^ Department of Mechanical Engineering City University of Hong Kong Hong Kong 999077 China; ^5^ City University of Hong Kong Matter Science Research Institute (Futian) No. 3, Binglang Road, Futian District Shenzhen 518045 China; ^6^ Department of Mechanical Engineering The University of Hong Kong Hong Kong 999077 China; ^7^ Inter‐University 3D Atom Probe Tomography Unit Department of Mechanical Engineering City University of Hong Kong Hong Kong 999077 China; ^8^ School of Materials Science and Engineering Dalian University of Technology Dalian 116024 China; ^9^ Department of Materials Science and Engineering The University of Tennessee (UT) Knoxville TN 37996 USA; ^10^ City University of Hong Kong Shenzhen Research Institute Greater Bay Joint Division Shenyang National Laboratory for Materials Science Shenzhen 518057 China; ^11^ Hong Kong Branch of National Precious Metals Material Engineering Research Center City University of Hong Kong Hong Kong 999077 China

**Keywords:** eutectic multi‐principal element alloys, mechanical property, phase‐field simulation, vermicular eutectic structure

## Abstract

Eutectic multi‐principal element alloys (EMPEAs), with multiple main elements in compositions and eutectic microstructures, are considered promising high‐performance materials for structural applications. The microstructure of EMPEAs usually exhibits a mixture of soft and hard phases in straight rod‐like or lamellar morphology, which contribute to a balanced synergy of strength and ductility. However, such conventional morphology may also constrain the possible space for further improving their mechanical properties, and the question proposed is whether the straight morphology can be kinked to unlock a new space for achieving better mechanical properties. Here an (AlCrFe_2_)_65_Ni_35_ EMPEA featuring an unseen kinked vermicular eutectic microstructure is successfully prepared. This innovative microstructure imparts remarkably improved strength‐ductility synergy to the EMPEA, which surpasses both its coarse‐grained counterpart and typical EMPEAs with straight morphologies, indicating a pronounced strengthening of the vermicular eutectic microstructure. The phase‐field simulation reveals the formation of such microstructure as the lack of crystallographic locking caused by the similar elastic modulus of the two eutectic phases. The findings not only expand the family of possible eutectic microstructures but also offer a pioneering paradigm for enhancing EMPEAs, paving the way for their application in high‐performance structural materials.

## Graphical abstract

Eutectic multi‐principal element alloys (EMPEAs) typically exhibit rod‐like or lamellar microstructures, constraining further mechanical improvements. This study introduces a novel (AlCrFe_2_)_65_Ni_35_ EMPEA with a unique vermicular microstructure, achieving unprecedented strength‐ductility synergy. Phase‐field simulations attribute this morphology to weak interphase anisotropy from matched elastic moduli, expanding design possibilities for high‐performance structural alloys.

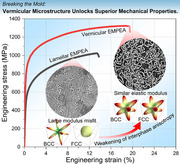

## 1 Introduction

The quest for advanced eutectic alloys with combined high strength and ductility is imperative for diverse industrial applications.^[^ ^1^, ^2^ ^]^ Recently, eutectic multi‐principal element alloys (EMPEAs) have emerged at the forefront of this research field as a promising class of eutectic alloys. The EMPEAs are characterized by compositions of multiple main elements, which offer a vast compositional space for exploring alloys with better mechanical performance like other medium‐entropy or high‐entropy alloys,^[^ ^3^, ^4^, ^5^, ^6^, ^7^, ^8^, ^9^, ^10^, ^11^ ^]^ and eutectic microstructure,^[^ ^12^, ^13^ ^]^ which is usually a mixture of soft face‐centered‐cubic (FCC) and hard body‐centered‐cubic (BCC) phases in straight rod‐like or lamellar morphology, contributes to a balanced synergy of high strength and ductility.^[^ ^14^ ^]^ At present, EMPEAs with excellent properties and extensive research are represented by the AlCoCrFeNi_2.1_,^[^ ^14^, ^15^, ^16^ ^]^ Al_19_Co_20_Fe_20_Ni_41_,^[^ ^13^ ^]^ Ni_40_Co_20_Fe_10_Cr_10_Al_18_W_2_,^[^ ^17^, ^18^ ^]^ and Al_18_Co_30_Cr_11_Fe_11_Ni_30_^[^ ^19^ ^]^ alloy systems. The EMPEAs have not only competitive room/high‐temperature mechanical properties but also have high melt flow and castability similar to eutectic alloys^[^ ^20^ ^]^ and additive manufacturability.^[^ ^15^, ^21^, ^22^ ^]^ This combination of properties and formability makes EMPEAs an attractive choice for structural materials in industrial applications.^[^ ^15^ ^]^

In recent years, many methods have been applied to further optimize the mechanical properties of EMPEAs, such as the addition of alloying elements,^[^ ^23^ ^]^ additive manufacturing,^[^ ^15^, ^22^, ^24^ ^]^ directional solidification,^[^ ^13^ ^]^ powder metallurgy,^[^ ^25^ ^]^ high‐pressure and high‐temperature treatment,^[^ ^26^ ^]^ and phase‐selective recrystallization.^[^ ^19^, ^27^ ^]^ Notably, the lamellae and rods almost always remain relatively straight after the application of these methods. The straight morphology originates from the competitive growth of the lamellae, which are locked into preferred crystallographic orientations during solidification due to the interphase anisotropy between eutectic phases.^[^ ^28^ ^]^ However, if the straight morphology of EMPEAs could be kinked to form a vermicular microstructure, which was not achieved, additional phase boundaries in various directions could be introduced to pin the dislocations. This new eutectic morphology feature would help the soft FCC phase to bear more deformation and thus take full advantage of the strain‐hardening capacity of all phases, thereby enhancing the strength and ductility. Therefore, in contrast to what has so far been experimentally reported and theoretically modeled, the realization of EMPEAs with the novel vermicular microstructure is expected to hold great potential for further improving the mechanical properties of eutectic alloys.

Here, we report an (AlCrFe_2_)_65_Ni_35_ EMPEA with a eutectic microstructure composed of a vermicular FCC phase embedded in a decomposed BCC matrix in the as‐cast state. This EMPEA has excellent mechanical properties that surpass those of typical EMPEAs. The formation mechanism of this microstructure is then revealed by a phase‐field simulation. These findings broaden the family of eutectic microstructures and are of great importance for academic study and their applications.

## 2 Results and Discussion

### 2.1 Composition Design and Microstructural Characterization

**Figure 1:**
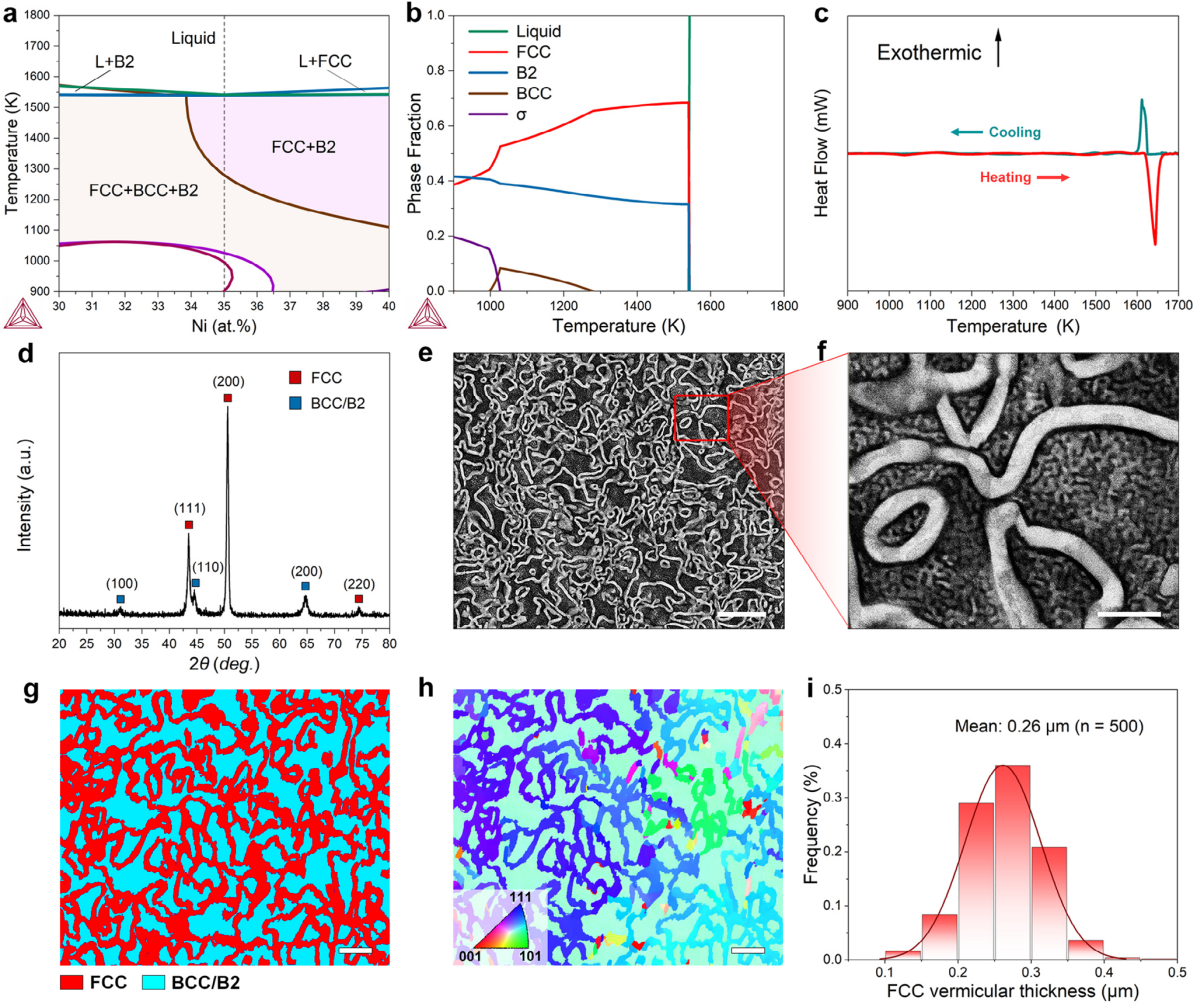
Phase diagram calculation, phase analysis, and microstructural characterization of the novel EMPEA. a, b): Pseudo‐binary phase diagram and vertical‐section phase diagram calculated using the CALPHAD method; c): DSC analysis reveals a single endothermic/exothermic peak during heating and cooling, suggesting simultaneous melting/solidification of the FCC and BCC phases; d): XRD analysis of the alloy confirms its FCC‐BCC dual‐phase structure, with the (100) peak indicating the presence of a B2 phase; e, f): SEM images display a vermicular structure, distinct from traditional lamellar eutectic alloys; g): EBSD phase map, showing phase proportions of 58.8% FCC and 41.2% BCC/B2 (volume percent); h): EBSD inverse pole figure; i): Histograms of the FCC thickness in the vermicular EMPEA. Scale bars, 5 µm in (e); 1 µm in (f); 2 µm in (g, h).

The eutectic composition of the (AlCrFe_2_)_65_Ni_35_ EMPEA is designed with the assistance of the CALPHAD (CALculation of PHAse Diagrams) method. The thermodynamic equilibrium pseudo‐binary phase diagram of the (AlCrFe_2_)_100‐_ *_x_* Ni*_x_* alloy system is calculated as shown in **Figure** **1**a, illustrating that the EMPEA would become eutectic when the mole fraction of Ni reached 35 atomic percent (at.%). The phases and phase fractions of the (AlCrFe_2_)_65_Ni_35_ EMPEA are also calculated, as presented in Figure 1b, which shows that a mixture of ≈68% FCC and ≈32% ordered BCC (B2) eutectic microstructure would form during solidification, and the fraction of the FCC matrix would further decrease with decreasing temperature. Furthermore, the values of the thermophysical parameters of the designed EMPEA comply with empirical criteria and tend to form an FCC+BCC dual‐phase microstructure,^[^ ^29^, ^30^ ^]^ which agrees with the CALPHAD result (Note S1, Supporting Information). Compared with binary eutectic alloys, this alloy shows a narrow temperature gap of ≈2.7 K between the liquidus and solidus temperatures (Figure S1, Supporting Information). This gap is allowed by the Gibbs’ phase rule as there are four elements in the EMPEA, and thus the liquid, FCC, and B2 phases can co‐exist within a temperature range. The calculation result further implies that a BCC phase would appear below 1280 K, and a sigma phase would precipitate below 1026 K, although the latter may be prohibited dynamically due to its low formation temperature.

To experimentally validate the CALPHAD predictions regarding the designed (AlCrFe_2_)_65_Ni_35_ EMPEA, this alloy is prepared by arc‐melting, followed by copper‐mold suction casting, and characterized. To confirm that the alloy is eutectic, the differential scanning calorimetry (DSC) analysis is performed. The alloy shows only one narrow endothermic/exothermic peak corresponding to melting/solidification during heating/cooling (Figure 1c), respectively, which provides evidence that the alloy is eutectic as designed. The discrepancy between the endothermic and exothermic peak temperatures is assumed to be caused by the temperature lag effect in DSC^[^ ^31^ ^]^ and the undercooling during solidification.^[^ ^32^ ^]^ The melting point is slightly higher than the eutectic temperature calculated by the CALPHAD method, which may indicate an incomplete thermodynamic database for the complex EMPEAs.

Next, the microstructure of the designed (AlCrFe_2_)_65_Ni_35_ EMPEA is characterized. Figure 1d–f show the X‐ray diffraction (XRD) pattern and scanning electron microscopy (SEM) images of the prepared EMPEA. These images indicate that the microstructure is composed of a disordered FCC phase (*a* = 3.598 Å), and a B2/BCC matrix (*a* = 2.881 Å, and the weak (100) diffraction peak proves the existence of an ordered BCC crystal structure (B2)). The eutectic structure of the present alloy has a unique vermicular morphology, which was not found in EMPEAs. Then, the crystal orientation and phase distribution of the eutectic structure is characterized by electron backscattering diffraction (EBSD) phase map and inverse pole figure, confirming the vermicular FCC phase and the BCC matrix (Figure 1g,h). These experimental results agree with the phases predicted by the CALPHAD method. The thickness of the vermicular FCC phase is ≈0.26 µm (Figure 1i), which is only ≈10% of the thickness of the FCC lamellar in traditional homologous EMPEAs.^[^ ^14^, ^16^, ^22^ ^]^ Moreover, it is found that the thickness can be reduced by increasing the cooling rate, as realized by casting the EMPEA in a stepped cylindrical mold with a gradually decreasing diameter (Note S2, Supporting Information). This trend also suggests that the vermicular microstructure is stable within a range of cooling rates.

**Figure 2:**
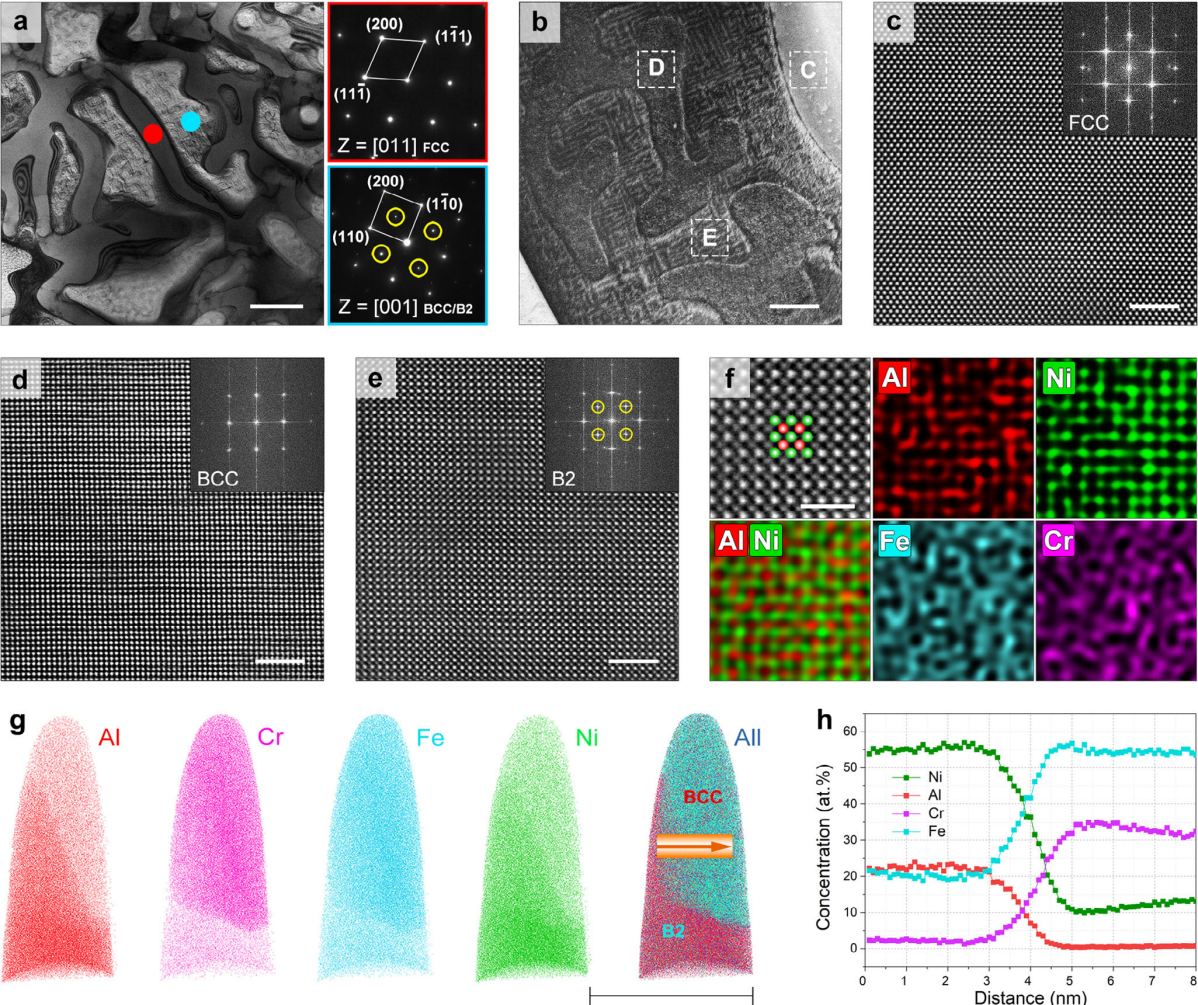
Multi‐scale analysis of the microstructure and elemental distribution of the vermicular EMPEA. a): BF‐STEM image of the FCC‐BCC phases and the corresponding SAED patterns. Superlattice points (marked by yellow circles) are observed along the [001]_BCC_ zone axis, indicating an ordered structure in the BCC‐phase area; b): BF‐STEM image of the BCC‐phase area shows a phase decomposition; c–e): Atomic‐scale aberration‐corrected STEM‐HAADF images of FCC, BCC, and B2 phases along the [011]_FCC_, [001]_BCC_, and [001]_BCC_, zone axes (areas labeled “C”, “D”, and “E” in b), inset: corresponding FFT patterns; f): Atomic structure of a B2 phase along the [001]_BCC_ zone axis, and the corresponding atomic‐resolution EDS mapping; g, h): Results of APT analysis performed on the phase boundary of the BCC and B2 phases. Scale bars, 1 µm in (a); 100 nm in (b); 2 nm in (c–e); 1 nm in (f); 50 nm in (g).

The microstructure is further analyzed by scanning transmission electron microscopy (STEM) and selected area electron diffraction (SAED) (Figure 2a,b). The vermicular FCC phase is confirmed by its typical FCC diffraction pattern, while the decomposed BCC matrix contains a typical B2 diffraction pattern, which confirms the existence of the B2 phase. Figure 2c–e exhibit the aberration‐corrected high‐angle annular dark‐field (HAADF) images of the atomic structure of the FCC, BCC, and B2 phases (corresponding to the c, d, and e areas in Figure 2b), respectively. Consistent with the SAED results, the corresponding fast Fourier transform (FFT) patterns confirm that the alloy consists of a vermicular FCC phase and decomposed BCC and B2 phases. The boundary between the disordered BCC and B2 phases is found to be coherent in the STEM‐HAADF image (Figure S2a, Supporting Information), where the orderedness is confirmed by the presence of the (100) pattern in the FFT image (indicated by the yellow circle in Figure S2c, Supporting Information). The existence of a coherent boundary is assumed to be caused by the very similar lattice parameters of the two phases (BCC: *a* = 2.876 Å; and B2: *a* = 2.881 Å), which also explains the overlap of their peaks in XRD, given the limited XRD resolution.

The compositions of the phases are investigated by STEM‐energy‐dispersive X‐ray spectroscopy (STEM‐EDS) mapping (Figure S3, Supporting Information), and the results show that the FCC phase is rich in Fe, Cr, and Ni, the BCC phase is rich in Fe and Cr, and the B2 phase is rich in Al and Ni, which is in agreement with the CALPHAD results (Table S1, Supporting Information). The chemical composition of the B2 phase is analyzed by atomic‐resolution EDS mapping (Figure 2f). The EDS map shows that the B2 phase exhibits an ordered structure with an Al‐rich sublattice and a Ni‐rich sublattice,^[^ ^33^ ^]^ which directly confirms the orderness. To further analyze the decomposed matrix, atom probe tomography (APT) analysis is performed on the phase boundary of the BCC and B2 phases, and the result confirms the compositional differences between the two phases (Figure 2g,h). Based on the above experimental results, it can be concluded that this (AlCrFe_2_)_65_Ni_35_ EMPEA is eutectic, and its microstructure is composed of a vermicular FCC embedded in a decomposed BCC + B2 matrix.

### 2.2 Mechanical Property

**Figure 3:**
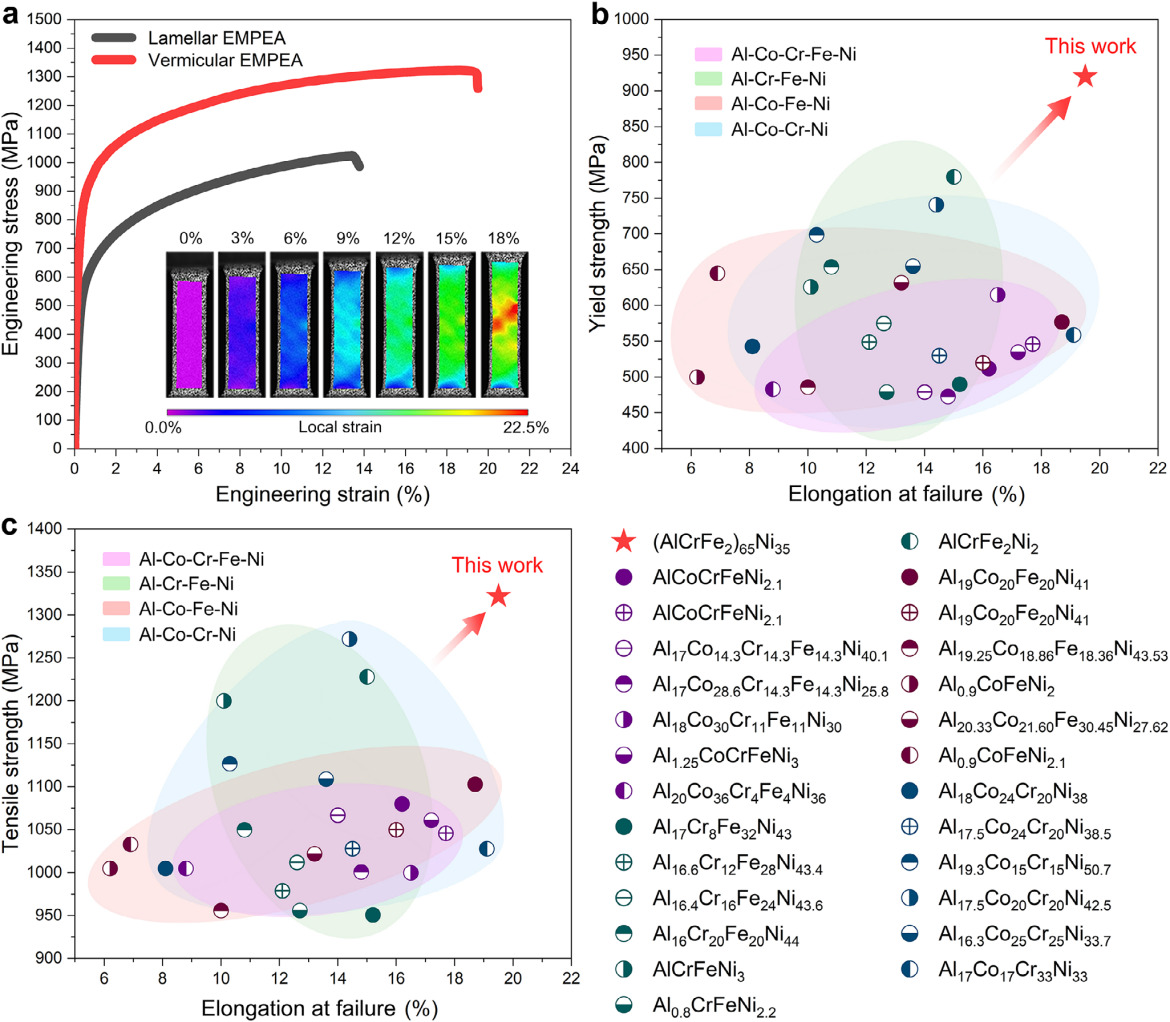
Analysis of room‐temperature tensile mechanical properties of the vermicular and lamellar EMPEAs. a): Engineering stress‐strain curves at room temperature for the vermicular EMPEA and lamellar EMPEA (AlCoCrFeNi_2.1_, at.%), demonstrating the remarkable strength‐ductility combination of the vermicular EMPEA. The inset shows a 2D tensile local strain map along the tensile direction (ε_*yy*_) for the vermicular EMPEA, created by DIC, revealing uniform deformation exceeding 15%; b, c): Comparison of uniform elongation and yield strength/ tensile strength between the vermicular EMPEA and Al‐Co‐Cr‐Fe‐Ni system lamellar EMPEAs, indicating significant mechanical‐performance advantages of the present alloy. The detailed data can be found in Table S2 (Supporting Information).

To evaluate the effect of the vermicular microstructure on the mechanical properties of the designed alloy, uniaxial tensile tests are conducted on the as‐cast samples. As shown in **Figure** **3**a, the vermicular EMPEA presents a yield strength of 920 MPa, a tensile strength of 1322 MPa, and an elongation at failure of 19.5%. This vermicular EMPEA shows better strength‐ductility synergy than most typical EMPEAs with straight lamellar microstructures, including the typical lamellar EMPEA AlCoCrFeNi_2.1_, as exhibited in Figure 3b, c, which indicates that the vermicular microstructure is beneficial to the mechanical properties.^[^ ^13^, ^14^, ^20^, ^34^, ^35^, ^36^, ^37^, ^38^, ^39^, ^40^, ^41^, ^42^, ^43^, ^44^, ^45^, ^46^, ^47^, ^48^, ^49^ ^]^ Additionally, we calculate the origin of the yield strength of the vermicular and lamellar EMPEAs (Note S3, Supporting Information), indicating that the tightly spaced phase boundary of the vermicular EMPEA has a significant contribution to the ultra‐high yield strength (≈39%).

**Figure 4:**
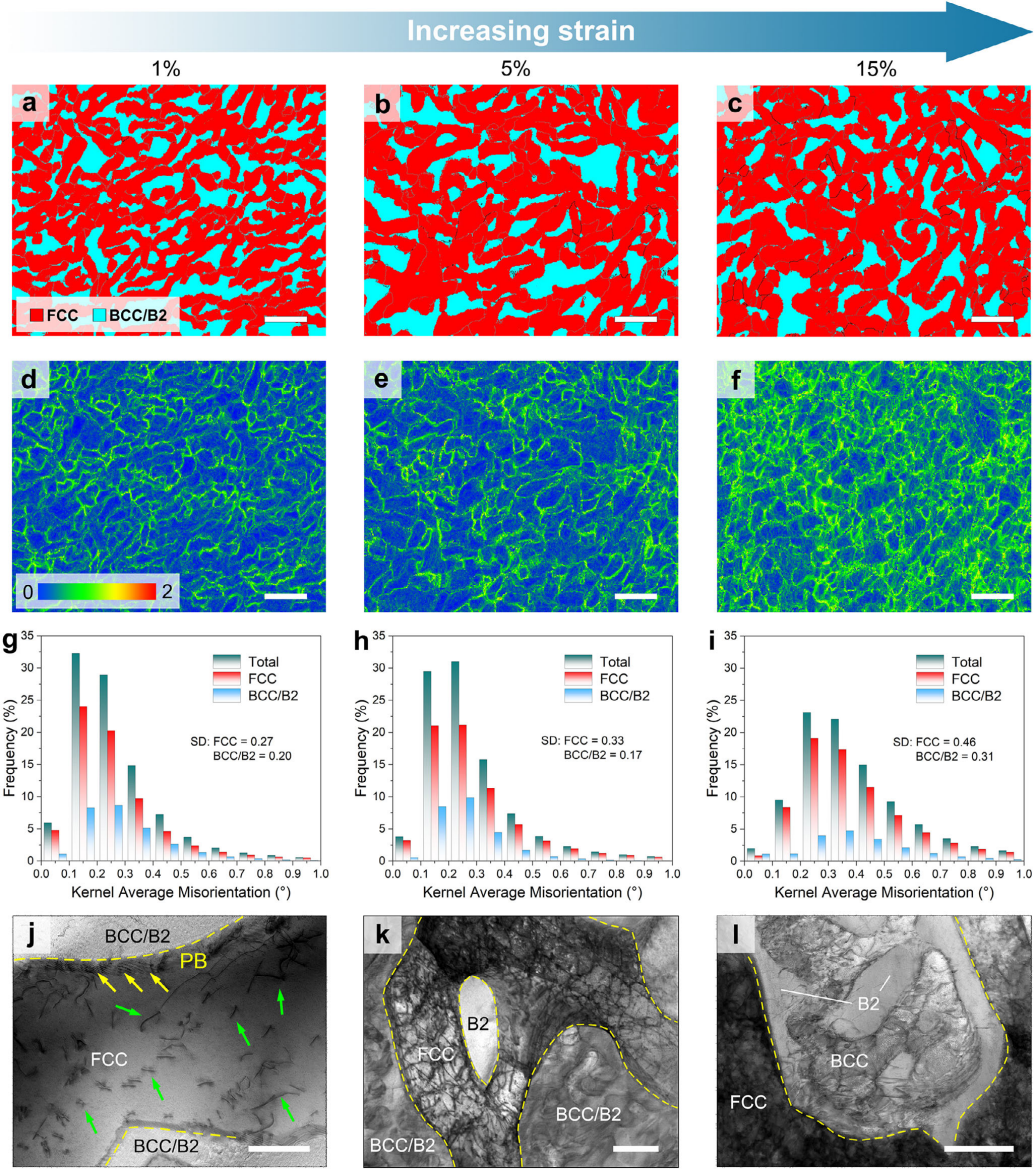
Meso‐ and micro‐scale deformation structures. a–c): EBSD phase maps of the vermicular EMPEA revealing the local misorientation at tensile strains of ≈1% (a), 5% (b) and 15% (c); d–f): KAM maps of the vermicular EMPEA revealing the local misorientation at tensile strains of ≈1% (d), 5% (e) and 15% (f); g–i): KAM misorientation statistics of the vermicular EMPEA at tensile strains of ≈1% (g), 5% (h) and 15% (i); j): TEM‐BF images of the 1% deformed sample: show that the plastic deformation at FCC‐B2 phase boundaries and within the FCC phase; k, l): BF‐STEM images depicting the FCC‐BCC/B2 phase deformed after different strains, 5% (k) and 15% (l). Scale bars, 2  µm in (a–f); 100  nm in (j, k); 200  nm in (l).

To investigate the mechanism of its excellent mechanical properties, the EBSD phase map, Kernel average misorientation (KAM) map, and TEM analysis are applied to the deformed samples (**Figure** **4**). After 1% strain, misorientation is observed in the vermicular FCC phase and the kink phase boundaries (PBs), indicating that early deformation occurred in the FCC phase with the formation of dislocations, and the PBs function as the barrier of the dislocations (Figure 4a,d,g). The accumulated dislocations near the PB are discovered by bright‐field STEM (BF‐STEM) image at the FCC‐B2 PB (highlighted by yellow arrows in Figure 4j). A small number of dislocations within the FCC phase are also observed (green arrows in Figure 4j), which is consistent with the EBSD‐KAM results. After 5% strain, the level of misorientation at the PBs and in the FCC phase further increases (Figure 4b,e,h). It can be observed from the BF‐STEM image that a high density of dislocations is observed in both the FCC and PB (Figure 4k), indicating that the FCC phase has undergone uniform and extensive plastic deformation. After 15% strain, the level of misorientation in the FCC phase continues to increase, and misorientation with high levels is also produced in the BCC/B2 phase (Figure 4c,f,i), indicating that significant plastic deformation has occurred in the BCC/B2 phase. The detailed deformed microstructures of the BCC and B2 phases are depicted in the BF‐STEM image in Figure 4l, which shows the presence of dislocations in both phases, indicating that both the BCC and B2 phases have undergone plastic deformation. The cross‐phase dislocations indicate that the adjacent FCC, B2, and BCC phases can partially co‐deform to release mechanical energy and reduce the chance of phase‐boundary decohesion or cracking.^[^ ^13^ ^]^ However, the TEM and KAM results show that the soft FCC phase bears significantly more plastic‐strain deformation than the other phases, as indicated by its notably higher dislocation density, while the hard BCC and B2 phases function as the strengthening phase by preventing the free deformation of the FCC phase. Therefore, most of the dislocations in the FCC phase can be considered geometrically necessary dislocations (GNDs).^[^ ^50^, ^51^, ^52^, ^53^, ^54^ ^]^ Further, these GNDs form a dislocation network and pile up at phase boundaries, and can interact with mobile dislocations, triggering a strong back stress‐strengthening effect.^[^ ^50^, ^55^, ^56^ ^]^ This combination is also observed in typical lamellar EMPEAs and is considered a key factor for achieving high strength‐ductility synergy.^[^ ^13^, ^34^ ^]^ Compared with the typical lamellar or rod‐like eutectic microstructure, the vermicular shape of the FCC phase decreases its micro‐scale shape anisotropy and introduces additional phase boundaries in various directions as observed in the KAM results, which helps the FCC phase deforming more homogeneously and bear greater deformation. Thus, the deformation is better coordinated, and the full advantage is taken of the strain‐hardening capacity of all phases,^[^ ^27^ ^]^ thereby increasing the strength‐ductility synergy. To better understand the stress distribution in deformed vermicular and lamellar eutectic structures, phase‐field microelasticity (PFM) simulations are performed. The ratio of average stress between the BCC and FCC phases is calculated for different microstructures (details in Note S4, Supporting Information). The results demonstrate that the vermicular eutectic structure significantly alleviates stress concentration (Figure S4, Supporting Information). This phenomenon can be attributed to the closer elastic moduli of the FCC and BCC phases in the vermicular EMPEA (Figure S5, Supporting Information), as well as the increased PB density, which facilitates stress redistribution. We propose that the more homogeneous stress distribution may promote co‐deformation of the FCC and BCC/B2 phases. This effect may also be shown by the comparable work‐hardening rates of the vermicular EMPEA and the typical lamellar AlCoCrFeNi_2.1_ EMPEA. The stronger back‐stress strengthening effect is also observed in cyclic loading‐unloading‐reloading experiments, where the vermicular EMPEA back‐stress increases with strain (as quantitatively depicted in Figure S6, Supporting Information), reaching 850 MPa near failure (43% higher than the lamellar AlCoCrFeNi_2.1_ EMPEA), indicating the alloy's significant hetero‐deformation‐induced strengthening.

To further confirm the relationship between the mechanical properties and the vermicular microstructure, the as‐cast sample is heat‐treated for 1 h at 1473 K to eliminate the vermicular microstructure. The heat‐treated sample shows a typical coarse dual‐phase microstructure with no obvious vermicular microstructure, as shown in Figure S7 (Supporting Information). Its yield strength and tensile strength are significantly reduced to 538 MPa (a 41.5% drop) and 998 MPa (a 24.5% drop), respectively, and a slight increase in the ductility is observed (19.5%–24%, a 23.1% increase). The decrease in the overall strength‐ductility synergy is evidence of the beneficial effect of the vermicular microstructure. In short, the vermicular microstructure gives the EMPEA excellent mechanical properties compared with typical EMPEAs.

### 2.3 Phase‐Field Simulation of the Vermicular Microstructure

**Figure 5:**
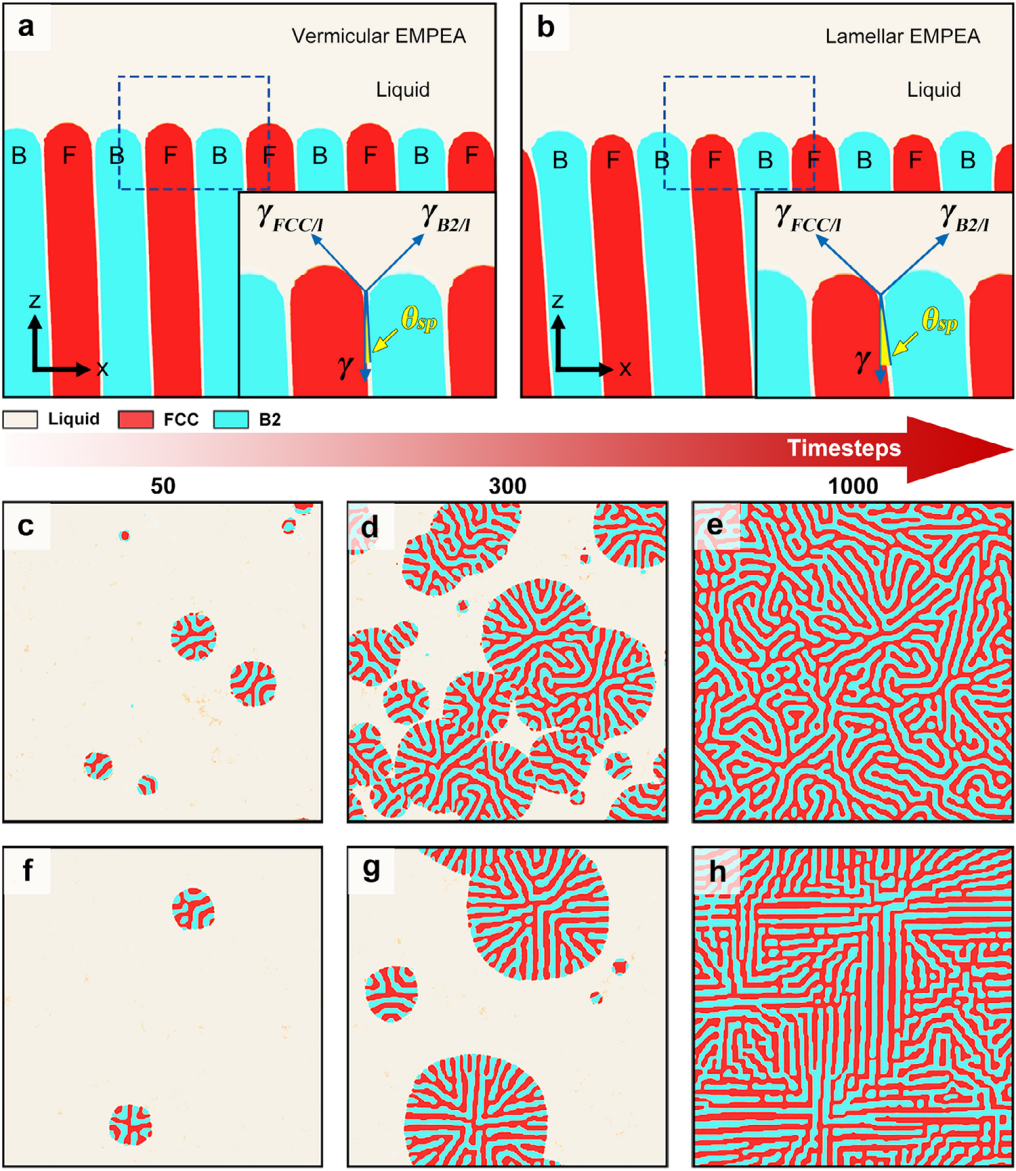
Simulated results for vermicular and lamellar EMPEAs. a): Young‐Herring equilibrium of vermicular EMPEA; the blue box presents an enlargement of the surface‐tension balance at the trijunction point, where θ_*sp*_indicates the tilt angle between the γ and the interphase boundary; b): Young‐Herring equilibrium of lamellar EMPEA and an enlargement of the surface‐tension balance at the trijunction point; c–e): Simulated nucleation and eutectic growth of vermicular EMPEA at 50, 300, and 1000 timesteps; f–h): Simulated nucleation and eutectic growth of lamellar EMPEA at 50, 300, and 1000 timesteps.

To investigate the formation mechanism of the vermicular microstructure, a time‐evolved phase‐field (PF) model combined with the PFM method is applied to simulate the eutectic growth process, the details of which can be found in the Experimental Section. We start with the simulation of FCC and B2 lamellar eutectic growth, while the BCC phase is not observed to precipitate during solidification, as shown in **Figure** **5**a. Recent theoretical and experimental studies have shed light on the significant influences of solid–solid interfaces on eutectic growth, where eutectic patterns exhibit strong dependence on the anisotropy of the solid–solid interface energy.^[^ ^28^, ^57^ ^]^ Generally, these anisotropies of energy are caused by differences between the crystallographic structures and orientations of eutectic phases.^[^ ^58^, ^59^ ^]^ Such elastically and structurally inhomogeneous systems can be characterized by the differences in elastic moduli and the crystal lattice misfit derived from PFM models.^[^ ^60^, ^61^ ^]^ The elastic‐modulus tensors of FCC and B2 phases are calculated based on the density functional theory (details in Note S5, Supporting Information), and the lattice misfit is obtained with the formula of ε0=2|aFCC−aB2|/|aFCC−aB2|(aFCC+aB2)(aFCC+aB2), where *a*_FCC_ and *a*_B2_ are the lattice constants of FCC and B2 phases measured from the XRD results. These parameters are used to simulate the local equilibrium at the trijunction points with a uniaxial thermal gradient in the direction, where the system strain energy at the solid–solid interface is explicitly captured based on the Khachaturyan–Shatalov (KS) theory.^[^ ^60^, ^62^ ^]^ Here, the equilibrium condition is imposed via the surface‐tension balance following the Young–Herring equation as:

(1) $$\gamma_{FCC/l}+\gamma_{B2/l}+\gamma =0$$

where γ_*FCC*/*l*_ and γ_*B*2/*l*_ are the surface tensions of the FCC‐liquid and B2‐liquid interfaces, respectively, and γ is the surface free energy of the FCC‐B2 boundary. In isotropic interface‐energy systems, the eutectic lamellae grow at a constant velocity, because γ are aligned with the growth axis, *z*. However, the eutectic lamellae may drift along the growth front due to the presence of interphase anisotropy, which in turn changes the balance of surface tensions at the trijunction points. This modified equilibrium leaves a tilt angle, θ_*sp*_ between the solid–solid interface and *z* in the steady state. For the vermicular EMPEA, negligible lateral drifting is observed at the trijunction points with a small lamellar tilt angle, θ_*sp*_, of ≈1°. It follows that the FCC/B2 interfaces are nearly parallel to the growth direction due to thermodynamic effects, indicating a weak interphase‐boundary anisotropy in the vermicular EMPEA. Our model reveals that the elastic moduli of the FCC and B2 phases for vermicular EMPEA are very close and interact nearly isotropically, as shown in Figures S5a–c (Supporting Information), which allows the lamellae to twist smoothly due to the presence of thermodynamic instability in the environment. Such growth instabilities of the orientations lead to the formation of vermicular lamellae. For comparison, a similar equilibrium simulation is also performed on the lamellar AlCoCrFeNi_2.1_ EMPEA, which is a typical EMPEA with an FCC and B2 lamellar eutectic microstructure. In contrast to the previous case, a large modulus misfit exists in the lamellar EMPEA as shown in Figures S5d–f (Supporting Information), causing a significant interface anisotropy and leaving a larger tilt angle, θ_*sp*_, of ≈6° at the trijunction points (see Figure 5b). Therefore, the eutectic lamellae prefer to grow steadily along a direction tilted with respect to *z* and form a lamellar eutectic microstructure, referred to as the crystallographic locking effect.^[^ ^58^ ^]^ Besides, the crystal lattice misfits for the vermicular and lamellar EMPEAs are 0.2230 and 0.2236, respectively. Due to the negligible difference between these values, misfit is not considered the main reason for the differences in the microstructures.

Furthermore, isothermal PF simulations of the solidification of the eutectic microstructures are performed for these two alloys to investigate the formation of their microstructures. In the simulations, the initial condition of the computational domain is set as a homogeneous metastable liquid with a non‐dimensional supercooling temperature, Δ*T* = −0.34, and a non‐dimensional concentration, *c* = 0, representing the eutectic composition. The non‐dimensionalization of the PF model is performed, as described in the Experimental Section. Langevin noise is applied to the liquid phase, and nucleation events occur automatically due to the undercooled temperature and noise. For the vermicular EMPEA, the nuclei form and gradually grow from the metastable liquid at the initial stage (Figure 5c). Over time, the FCC and B2 phases grow in a twisted manner due to the influence of local thermal fluctuations (Figure 5d). Such perturbations, which result in unstable boundary orientations on the eutectic pattern,^[^ ^59^ ^]^ cannot be resisted due to the lack of crystallographic locking. Subsequently, the vermicular eutectic microstructure is observed when the solid volume fraction approaches unity (see Figure 5e), which is in good agreement with our experimental results (Figure 1e) and explains the formation of the vermicular eutectic microstructure.

For comparison, Figure 5f–h demonstrate the solidification process of the lamellar EMPEA under the same environmental conditions. As a consequence of the interphase anisotropy, preferred orientations of lamellae are observed during growth (see Figure 5g) due to the significant crystallographic locking effect against the local thermal fluctuations, and therefore, the boundary instability of lamellae is suppressed. After solidification, typical lamellar morphologies of FCC and B2 phases are observed with presumably locked orientations, as illustrated in Figure 5h. These simulated steady patterns are consistent with the experimental findings (Figure S8, Supporting Information).^[^ ^63^, ^64^ ^]^ In short, dynamic simulations by the PF model reveal that the weak interphase anisotropy weakens crystallographic locking and results in a vermicular growth manner.

The experimental findings on crystallographic orientation relationships provide partial yet critical validation of the phase‐field simulation results (details in Note S6, Supporting Information). Specifically, the semi‐coherent K–S orientation relationship (Figure S9a–d, Supporting Information) observed in lamellar EMPEAs confirms the existence of a crystallographic locking effect at the FCC/B2 interface. This locking effect is beneficial for promoting the anisotropic growth of FCC/B2 lamella. The lack of crystallographic locking in vermicular eutectic (Figure S9e–h, Supporting Information) experimentally validates the simulation prediction that solute diffusion asymmetry dominates over crystallographic effects in this regime. Therefore, thermal fluctuations in the environment destabilize planar growth fronts.

We note that a similar vermicular microstructure has also been observed in the AlCrFe_2_Ni_2_ medium‐entropy alloy (MEA).^[^ ^43^, ^65^ ^]^ However, the AlCrFe_2_Ni_2_ MEA is not eutectic, and a thermodynamic calculation indicates that a pre‐eutectic FCC phase with an ≈35% phase fraction would form before the formation of the binary FCC + B2 eutectic phases during the solidification of the AlCrFe_2_Ni_2_ MEA (Figure S10, Supporting Information).^[^ ^66^ ^]^ Instead, FCC Widmanstätten^[^ ^67^, ^68^ ^]^ or other microstructures^[^ ^65^, ^67^, ^69^ ^]^ would form and be observed, while the vermicular microstructure may not appear. In this study, the (AlCrFe_2_)_65_Ni_35_ EMPEA is eutectic, which stabilizes the vermicular microstructure so that it can be observed under various cooling rates (Figure S11, Supporting Information).

## 3 Conclusion

In summary, the (AlCrFe_2_)_65_Ni_35_ EMPEA is successfully developed with a unique vermicular FCC phase embedded within a BCC + B2 matrix. This innovative and distinct microstructure endows the (AlCrFe_2_)_65_Ni_35_ EMPEA with superior mechanical properties to those of most other EMPEAs with typical straight rod‐like or lamellar microstructures. The formation of the vermicular microstructure is explained by a PF simulation, which indicates that the key reason is the lack of crystallographic locking due to the weak interphase‐boundary anisotropy caused by the similar elastic moduli of the FCC and B2 phases. Moving forward, we believe the present work not only extends the frontier of eutectic‐microstructure possibilities but also establishes a novel paradigm to enhance EMPEAs with great capability for future applications as high‐performance materials. For future work, we aim to realize this vermicular microstructure in other eutectic alloys to enhance their mechanical properties for applications. We also aim to deepen the understanding of the role of the elements (e.g., the addition of other alloying elements) and preparation methods (e.g., additive manufacturing) in the microstructure and the mechanical properties, thereby flourishing this alloy family.

## 4 Experimental Section

### Alloy Design

The thermodynamic equilibrium phase diagram of the (AlCrFe_2_)_100‐_ *_x_* Ni*_x_* quaternary EMPEA and the phase fractions and compositions of the (AlCrFe_2_)_65_Ni_35_ were calculated by the CALPHAD approach.^[^ ^70^ ^]^ The pseudo‐binary phase diagram of the alloy system was calculated by the Thermo‐Calc 2021 software equipped with the TCHEA4 database.

### Alloy Preparation and Characterization

Al, Cr, Fe, and Ni metal ingots with a purity greater than 99.95 weight percent (wt.%) were proportionally mixed and arc‐melted under a high‐purity argon‐gas atmosphere using a vacuum arc‐melting equipment. To increase the homogeneity of the produced alloy, the melting process was conducted five times. Subsequently, alloy ingots with a size of 70 mm × 10 mm × 10 mm were prepared using the copper‐mold suction casting method. Employing electrical‐discharge machining (EDM), the alloy was cut into various shapes, ground using metallographic sandpapers up to 4000 grits, and polished with 0.05 and 0.02 µm SiO_2_ suspension polishing solutions before characterization.

The enthalpies of endothermic and exothermic reactions of the as‐cast alloy were measured using DSC (Discovery SDT 650, TA Instruments, USA). The crystal structures of the alloy were investigated by means of X‐ray diffraction (XRD) with Cu‐Kα radiation (DX‐2700BH, Haoyuan Instrument Co., Ltd, Dandong, China). The scan range for the XRD test was 20°–80° with a step of 0.02°. The phase structure and morphological distribution of the alloy were observed by the field‐emission SEM (Helios 5 CX, Thermo Fisher Scientific, USA) equipped with an EBSD system (Velocity Super, EDAX, USA). To further characterize the microstructure of EMPEA, a field‐emission scanning transmission electron microscope (STEM, Tecnai G2 F20, Frequency Electronics, Inc., USA) with high‐resolution imaging, and SAED functions was applied at 200 kV. The elemental distributions of the different phases were analyzed using an EDS (Bruker, Germany) attached to the TEM. The preparation of TEM samples involved initially grinding and polishing the alloy to a thickness below 50 µm, followed by automatic electrolytic thinning using an electrochemical twin‐jet polisher (Tenupol‐5, Struers, Germany). Atomic‐structure observation of the FCC, BCC, and B2 phases using the aberration‐corrected TEM at 300 kV (AC‐TEM, Titan Themis, FEI, USA), and chemical characterization via compositional mapping was performed by Super‐X detectors (EDS energy resolution ≤ 136 eV for Mn‐Kα and 10 kcps (output)) with four windowless electric refrigeration energy spectrum probes. Thin sample slices (≈50 nm) for the AC‐TEM analysis were prepared by locating and lift‐outs with focused ion beam‐SEM (FIB‐SEM, Thermo Scientific Helios 5 CX, USA). To measure the mechanical properties of the alloy, the alloy ingot was processed by a wire EDM and computerized numerically controlled machining equipment into smooth‐surfaced, dog‐bone‐shaped tensile specimens with a length, width, and thickness of 20, 6, and 1 mm, respectively, and a gauge length of 8 mm (Figure S12, Supporting Information). These specimens were prepared for room‐temperature tensile testing, which was conducted on a universal testing machine (AGS‐X 10 KN, Shimadzu, Japan) with a loading rate of 0.3 mm min^−1^, and the strain‐measurement accuracy optimized using a video extensometer (RVX‐112B, Nanjing, China). Deformation areas were visualized using the digital image correlation (DIC) method (Vic Snap 9, Correlated Solutions, USA; data processing was performed using Vic‐2D 7). Needle‐shaped specimens required for the APT analysis were fabricated by lift‐outs and annular‐milled in an FEI Scios FIB‐SEM apparatus. The APT characterizations were performed in a local electrode atom probe (LEAP 5000 XR, Cameca, France). The specimens were analyzed at 70 K in voltage mode, a pulse repetition rate of 200 kHz, a pulse fraction of 20%, and an evaporation detection rate of 0.2% atom per pulse. The AP Suite 6.1 data analysis workstation was used to create the 3D reconstructions and perform data analysis.

### Phase‐Field Model and Phase‐Field Microelasticity Method

To simulate the solidification process, we employed a PF model coupled with the PFM method. This model characterized the solidification dynamics by capturing the solid–liquid and solid–solid interfaces between different phases, involving both the process thermodynamics and elastic inhomogeneities. To determine the solid–liquid and solid–solid interfaces, the real concentration field, *C*, was non‐dimensionalized to a scalar concentration field, $c(\vec{r},t)$, by c(r⃗,t)=(C−CE)/(C−CE)(CFCC−CB2)(CFCC−CB2), where *C_E_* is the eutectic composition, and *C_FCC_* and *C*_*B*2_ represent the equilibrium compositional values for the FCC and B2 phases, respectively. With this definition, the FCC and B2 phases were separated by positive and negative values, respectively $c(\vec{r},t)$, where $c(\vec{r},t)=0$ represent the eutectic compositions, which were mainly found in the liquid phase. Additionally, a non‐conserved order parameter, $\phi (\vec{r},t)$, was defined as representing the liquid‐solid field to describe solidification, where the sign $\phi (\vec{r},t)$ determines whether the phase is liquid (ϕ < 0) or solid (ϕ > 0), and $c(\vec{r},t)$ only separates in the solid region. These two order parameters were coupled through a Ginzburg‐Landau free energy functional *F*(ϕ, *c*), in the PF formulation, given as:

(2) $$F\left(\phi ,c\right)=\int_{V}\left[f\left(\phi ,c\right)+\frac{\kappa_{\phi }}{2}{\left(\nabla \phi \right)}^{2}+\frac{\kappa_{c}}{2}{\left(\nabla c\right)}^{2}\right]dV$$

where κ_ϕ_ and κ_*c*_ are the gradient energy coefficients with respect to order parameters. *f*(ϕ, *c*) denotes the free‐energy density functional and reflects the thermodynamic interactions of the liquid and eutectic phases (see details in Note S7, Supporting Information). The second and third terms were introduced to penalize the presence of sharp interfaces. This sharp‐interface formulation captured the solid–liquid interfaces by considering the local thermodynamic‐equilibrium diffusion and capillary effect collectively, and therefore, the growth front of lamellae accommodated the curvature at the solid–liquid interface, which was assumed to be isotropic.^[^ ^58^, ^71^ ^]^

To quantitatively model the eutectic solidification and the interfaces, the Allen–Cahn and Cahn–Hilliard equations were employed, in which the temporal evolution of the field variables, ϕ and *c*, is given by:

(3) $$\frac{\partial \phi }{\partial t}=-L\frac{\delta F}{\delta \phi }+\eta_{\phi }$$

(4) $$\frac{\partial c}{\partial t}=\nabla \left(M\nabla \left(\frac{\delta \left(F+F^{el}\right)}{\delta c}\right)\right)+\eta_{c}$$

respectively, where *L* denotes the mobility characterizing the liquid‐solid transition, and *MM* is the chemical mobility constant. η_ϕ_ and η_*c*_ denote the Langevin noises for $\phi (\vec{r},t)$ and $c(\vec{r},t)$, respectively. These noises were applied to the liquid phase to trigger nucleation in the PF model by introducing thermal fluctuations. *F^el^* denotes the elastic‐energy function and is implicit in the PFM model, enabling it to systematically implement the anisotropic interphase energy between solid phases.

The solid–solid interface energy and its anisotropy could be analytically characterized based on the elastic‐modulus mismatch and crystal‐lattice misfit between two adjacent phases according to the KS theory, which is conventionally applied to such structurally and elastically inhomogeneous systems and includes an explicit expression of the system's elastic energy.^[^ ^62^ ^]^ The anisotropy is implied by the system's elastic energy only at the solid–solid interface boundary, while at the solid–liquid interface, the anisotropy is negligible by assumption.^[^ ^72^ ^]^ Based on the KS theory, our model employed the PFM method to solve the system elastic‐energy function, *F^el^*, which involves the description of interface boundaries at the isotropic/anisotropic elastic equilibrium. Here, *F^el^* is expressed as follows:

(5) $$F^{el}=\int_{V}\frac{1}{2}C_{ijkl}\left(\varepsilon_{ij}-\varepsilon_{ij}^{*}\right)\left(\varepsilon_{kl}-\varepsilon_{kl}^{*}\right)dV$$

where *C_ijkl_* is the coordinate‐dependent elastic modulus and was calculated by $C_{ijkl}=C_{ijkl}^{p}\times c+C_{ijkl}^{m}\times \left(1-c\right)$. $C_{ijkl}^{p}$ and $C_{ijkl}^{m}$ are elastic‐modulus tensors of precipitate and matrix phases prescribed as FCC and B2 phases, respectively. ε_*ij*_ denotes the strain field, and $\varepsilon_{ij}^{*}$ is the eigenstrain. The strain field, ε_*ij*_, was calculated iteratively following the stress equilibrium equation, ∇σ_*ij*_ = 0, in which the stress, σ_*ij*_, is related to ε_*ij*_ by the linear Hooke's law, $\sigma_{ij}=C_{ijkl}\left(\varepsilon_{ij}-\varepsilon_{ij}^{*}\right)$.^[^ ^60^ ^]^ The eigenstrain, $\varepsilon_{ij}^{*}$, reflects the structural inhomogeneities corresponding to the crystal‐lattice misfit, ε^0^ and concentration $\varepsilon_{ij}^{*}=\varepsilon^{0}c\delta_{ij}$, where δ_*ij*_ is the Kronecker delta function. The numerical implementation of the model is given in Note S8 (Supporting Information).

## Conflict of Interest

The authors declare no conflict of interest.

## Supporting information



Supporting Information

## Data Availability

The data that support the findings of this study are available from the corresponding author upon reasonable request.
