# Supplementary material for: Vermicular Eutectic Multi‐Principal Element Alloy with Exceptional Strength and Ductility
Source: Adv Sci (Weinh). 2025 Apr 3;12(25):2501150. doi: 10.1002/advs.202501150 (PMC12225001; doi:10.1002/advs.202501150)
Supplement: Supplementary file 1 — Supporting Information [file ADVS-12-2501150-s001.docx]

Supplementary Information

**Vermicular Eutectic Multi-Principal Element Alloy with Exceptional Strength and Ductility**

Liufei Huang^1,2†^, Yicheng Han^3,4†^, Yaoning Sun^2^, A. S. L. Subrahmanyam Pattamatta^6^, Junhua Luan^7^, Qing Wang^8^, Congcong Ren^1^, Yuanfeng Zhou^1^, Jinfeng Li^1*^, Hengwei Luan^4,5*^, Peter K. Liaw^9^, and Jian Lu^3,4,10,11*^

^1^Institute of Materials, China Academy of Engineering Physics, Mianyang, China

^2^School of Mechanical Engineering, Xinjiang University, Urumqi, China

^3^CityU-Shenzhen Futian Research Institute, Shenzhen, China

^4^Department of Mechanical Engineering, City University of Hong Kong, Hong Kong, China

^5^City University of Hong Kong Matter Science Research Institute (Futian), No. 3, Binglang Road, Futian District, Shenzhen, China

^6^Department of Mechanical Engineering, The University of Hong Kong, Hong Kong, China

^7^Inter-University 3D Atom Probe Tomography Unit, Department of Mechanical Engineering, City University of Hong Kong, Hong Kong, China

^8^School of Materials Science and Engineering, Dalian University of Technology, Dalian, China

^9^Department of Materials Science and Engineering, The University of Tennessee (UT), Knoxville, TN, USA

^10^City University of Hong Kong Shenzhen Research Institute, Greater Bay Joint Division, Shenyang National Laboratory for Materials Science, Shenzhen, China

^11^Hong Kong Branch of National Precious Metals Material Engineering Research Center, City University of Hong Kong, Hong Kong, China

†: These authors contributed equally: Liufei Huang, Yicheng Han

*: Corresponding authors:

Jinfeng Li: lijinfeng305@126.com

Hengwei Luan: hengluan@um.cityu.edu.hk;

Jian Lu: [jian.lu@cityu.edu.hk](mailto:jian.lu@cityu.edu.hk)

**Contents**

Supplementary Notes

Supplementary Note 1. Empirical parameters and CALPHAD method of the designed EMPEA.3

Supplementary Note 2. Casting the EMPEA with different cooling rates.4

Supplementary Note 3. Yielding behavior of dual-phase EMPEAs.5

Supplementary Note 4. Stress distribution with PFM method.10

Supplementary Note 5. Calculation of elastic properties.12

Supplementary Note 6. EBSD crystallographic orientation analysis.14

Supplementary Note 7. Free energy density functional.15

Supplementary Note 8. Numerical implementation of the phase-field model.17

Supplementary Figures

Figure S1. Vertical cross-sectional phase diagram near the melting point calculated using the CALPHAD method.19

Figure S2. Atomic-scale image of the BCC–B2 phase boundary observed by aberration-corrected STEM.20

Figure S3. Phase structure and elemental distribution of vermicular EMPEAs.21

Figure S4. Distribution of stress in vermicular and lamellar EMPEAs under uniaxial tension in y direction.22

Figure S5. 3D representation of directional dependence of Young’s modulus for FCC and BCC phases in EMEPAs.23

Figure S6. Back-stress analysis of the lamellar and vermicular EMPEAs.24

Figure S7. Engineering stress–strain curves and phase structure of vermicular EMPEAs before and after solution treatment.25

Figure S8. SEM image of a typical lamellar EMPEA.26

Figure S9. EBSD analysis of lamellar and vermicular EMPEAs.27

Figure S10. Vertical cross-sectional phase diagram calculated on the AlCrFe_2_Ni_2_ alloy using the CALPHAD method.28

Figure S11. Vermicular microstructure observed under different cylindrical sample diameters.29

Figure S12. Detailed geometry of the dog-bone-shaped sample.30

Supplementary Tables

Table S1. Composition of phases at 1,250 K calculated by the CALPHAD method.31

Table S2. Detailed tensile properties of the vermicular EMPEA and Al-Co-Cr-Fe-Ni system lamellar EMPEAs.32

Table S3. Thermophysical parameters of the (AlCrFe_2_)_65_Ni_35_ EMPEA.34

Table S4. Parameters used in phase-field simulation.35

Table S5. Lattice constants and second-order elastic tensors.36

**References**37

Supplementary Notes

Supplementary Note 1. Empirical parameters and CALPHAD method of the designed EMPEA.

The strategy in this work aimed to explore low-cost EMPEA while maintaining competitive mechanical properties. Therefore, it is necessary to develop a novel eutectic composition to achieve high strength and ductility. We start by substituting Co with Fe in the well-known AlCoCrFeNi_2.1_ EMPEA of the Al-Co-Cr-Fe-Ni alloy system, and we get the AlCrFe_2_Ni_2.1_ MPEA. Then, several empirical parameters of the designed EMPEA were calculated to characterize the alloy phases, as shown in Table S3. Notably, the values of the thermophysical parameters of the designed EMPEA indicate that the alloy would form a dual-phase microstructure. Finally, in order to obtain a eutectic composition, the mole fraction of Ni is adjusted according to the thermodynamic equilibrium phase diagram of the (AlCrFe₂)_100-_*_x_*Ni*_x_* alloy system by the CALPHAD method. Figures 1(a and b) in the manuscript illustrate that the MPEA indeed would possess an eutectic structure when the mole fraction of Ni reaches 35 atomic percent (at.%).

Supplementary Note 2. Casting the EMPEA with different cooling rates.

The raw metal materials (purity greater than 99.95 wt.%) of the (AlCrFe_2_)_65_Ni_35_ EMPEA were mixed in proportion and remelted at least four times in an arc-melting furnace under an argon gas atmosphere to increase homogeneity. Then, the metal sample was suction-casted using a stepped cylindrical mold to prepare metal samples with gradually decreasing diameters. With decreasing the diameter, the cooling rate increased and the microstructure was gradually refined, as presented in Figure S11.

Supplementary Note 3. Yielding behavior of dual-phase EMPEAs

Usually, due to the differences in properties between FCC and BCC phases, the yield strengths of FCC-BCC dual-phase EMPEAs mainly come from the lattice-friction-resistance strength of the FCC phase and the dislocation propagation resistance at the FCC-BCC phase boundary [1, 2]. Therefore, we estimate the yield strength of lamellar and vermicular EMPEAs by calculating the intrinsic strength of the FCC phase ($\sigma_{FCC}$) [3, 4] and the phase-boundary-strengthening value ($\sigma_{PB}$) [2, 5]:

|  | $\sigma_{y}=\sigma_{FCC}+\sigma_{PB}$ | (1) |
| --- | --- | --- |

According to previous studies [3, 4], the intrinsic strength of the FCC phase in EMPEAs can be expressed according to Vegard’s law:

|  | $\sigma_{FCC}=\sum c_{i}\sigma_{i}$ | (2) |
| --- | --- | --- |

where, $c_{i}$ is the content of the *i*^th^ element, and $\sigma_{i}$ represents the resulting strengthening of the mismatch parameter introduced by the *i*^th^ element, which can be expressed as:

|  | $\sigma_{i}=AG\delta_{i}^{4/3}c_{i}^{2/3}$ | (3) |
| --- | --- | --- |

where *A* is a non-dimensional constant dependent on the alloys of 0.04 [3]. *G* is the shear modulus of the FCC alloy, which can be obtained according to Vegard’s law: $G=\sum c_{i}G_{i}$. $\delta_{i}$ is the mismatch-parameter introduced by the *i*^th^ element, which can be expressed as [3, 6]:

|  | $\delta_{i}=\xi(\Delta G_{i}^{2}+\beta^{2}\Delta r_{i}^{2})^{1/2}$ | (4) |
| --- | --- | --- |

where $\xi$ is a statistical function related to the dislocation type and slip system [6, 7]. $\xi$ is equal to 1 for FCC metals and 2.5 for BCC metals [3]. $\beta$ represents a parameter related to the dislocation type. Generally, the value range of screw dislocations is $3<\beta<16$, and that of edge dislocations is $\beta\geq16$ [6, 8]. Usually, the deformation of the FCC phase at room temperature is dominated by edge dislocations, so the value of $\beta$ here is 16 [6]. $\Delta G_{i}$ and $\Delta r_{i}$ are the elastic-modulus mismatch and atomic-size mismatch introduced by the *i*^th^ element, respectively, which can be expressed by Equation 5 and 6 [3]:

|  | $\Delta G_{i}=\frac{\Delta G_{ijklm/ijkl}^{ave}-\Delta G_{ijkl/ijk}^{ave}}{\Delta c_{i}}$ | (5) |
| --- | --- | --- |
|  | $\Delta r_{i}=\frac{\Delta r_{ijklm/ijkl}^{ave}-\Delta r_{ijkl/ijk}^{ave}}{\Delta c_{i}}$ | (6) |

where $\Delta G_{ijklm/ijkl}^{ave}$ and $\Delta r_{ijklm/ijkl}^{ave}$ are the average elastic mismatch and average atomic-size mismatch of the *ijklm/ijkl* alloys, respectively, which can be calculated by Equation 7 and 8 [3]:

|  | $\Delta G^{ave}=\sum_{i}^{n} \sum_{i}^{n} c_{i}c_{j}\Delta G_{ij}=(c_{1},c_{2},\cdots,c_{n})\left(\begin{matrix} \Delta G_{11} & \Delta G_{12} & \cdots& \Delta G_{1n} \\ \Delta G_{21} & \Delta G_{22} & \cdots& \Delta G_{2n} \\ \vdots& \vdots& \ddots& \vdots\\ \Delta G_{41} & \Delta G_{42} & \cdots& \Delta G_{4n} \end{matrix}\right)\left(\begin{matrix} c_{1} \\ c_{2} \\ \vdots\\ c_{n} \end{matrix}\right)$ | (7) |
| --- | --- | --- |
|  | $\Delta r^{ave}=\sum_{i}^{n} \sum_{i}^{n} c_{i}c_{j}\Delta r_{ij}=(c_{1},c_{2},\cdots,c_{n})\left(\begin{matrix} \Delta r_{11} & \Delta r_{12} & \cdots& \Delta r_{1n} \\ \Delta r_{21} & \Delta r_{22} & \cdots& \Delta r_{2n} \\ \vdots& \vdots& \ddots& \vdots\\ \Delta r_{41} & \Delta r_{42} & \cdots& \Delta r_{4n} \end{matrix}\right)\left(\begin{matrix} c_{1} \\ c_{2} \\ \vdots\\ c_{n} \end{matrix}\right)$ | (8) |

where $\Delta G_{ij}$ and $\Delta r_{ij}$ represent the elastic mismatch and atomic size mismatch between atoms, *i* and *j*, respectively, and are expressed by Equation 9 and 10 [3]:

|  | $\Delta G_{ij}=\frac{2\left\vert G_{i}-G_{j}\right\vert}{\left(G_{i}+G_{j}\right)}$ | (9) |
| --- | --- | --- |
|  | $\Delta\text{r}_{ij}=\frac{2\left\vert r_{i}-r_{j}\right\vert}{\left(r_{i}+r_{j}\right)}$ | (10) |

where $G_{i}$ and $G_{j}$ denote the shear moduli of pure metals, *i* and *j*, and $r_{i}$ and $r_{j}$ represent the atomic radii of *i* and *j* atoms. According to previous studies, the shear modulus and atomic radius for each atom for the present alloys are (Al, 26 GPa; Co, 75 GPa; Cr, 115 GPa; Fe, 82 GPa; and Ni, 76 GPa) and (Al, 0.143 nm; Co, 0.125 nm; Cr, 0.128 nm; Fe, 0.126 nm; and Ni, 0.124 nm), respectively [3]. The intrinsic strengths of the FCC phases of the vermicular and lamellar EMPEAs were calculated by Equation 2−10 to be ~ 560 MPa and ~ 453 MPa, respectively.

In the present alloy, the FCC-B2 heterogeneous interfaces also provide significant yield strength to the alloy by hindering the movement of dislocations. Generally, the grain-boundary or phase boundary strengthening effect can be expressed by the Hall-Petch relationship. In heterostructural EMPEAs, dislocations are pinned and accumulate at phase boundaries. When plastic deformation is dominated by dislocation accumulation, dislocations need to overcome tightly spaced interface barriers. Therefore, we consider the influence of the interface-barrier stress ($\tau_{PB}$). According to the continuum dislocation pile-up theory, the relationship between the phase-boundary strengthening ($\sigma_{PB}$) and interface-barrier stress ($\tau_{PB}$) can be expressed as follows [2, 5]:

|  | $\sigma_{PB}=kL^{-1/2}=\left(\frac{\tau_{PB}Gb\left(1-\nu\right)}{\pi L}\right)^{1/2}$ | (11) |
| --- | --- | --- |
|  | $\tau_{PB}=\tau_{\alpha}+\tau_{k}+\tau_{misfit}+\tau_{ch}$ | (12) |

where, $G$ is the shear modulus, $b=\sqrt{3}a_{FCC}/2$ is the Burgers vector of the active slip system in the incident grain [9], and $\nu$ is the Poisson’s ratio, estimated as 0.25 [10]. $L$ is the pile-up length (representing the thickness of the FCC phase of the present alloys, and the FCC phase thicknesses of vermicular and lamellar EMPEAs are ~ 270 nm and ~ 920 nm, respectively). $\tau_{\alpha}$ is the interface-independent barrier strength determined from the Hall−Petch relationship; $\tau_{K}$ is the contribution from Koehler stresses; $\tau_{misfit}$ is the strengthening caused by the lattice mismatch; and $\tau_{ch}$ is the strengthening induced by the difference in the stacking fault energies (SFE) between FCC and B2 phases. $\tau_{\alpha}$, $\tau_{K}$, $\tau_{misfit}$, and $\tau_{ch}$ were calculated by Equation 13−16, respectively [2, 11-13]:

|  | $\tau_{\alpha}=\frac{\pi k^{2}}{Gb\left(1-\nu\right)}$ | (13) |
| --- | --- | --- |
|  | $\tau_{K}=\frac{\left(G_{FCC}-G_{B2}\right)}{4\pi\left(G_{FCC}+G_{B2}\right)}\times\frac{G_{FCC}}{2}$ | (14) |
|  | $\tau_{misfit}=0.5\Delta G\left(\frac{2b\left(\delta-\varepsilon\right)}{\lambda}\right)^{1/2}$ | (15) |
|  | $\tau_{ch}=\frac{\Delta\gamma}{b}$ | (16) |

where $k$ is the Hall−Petch constant, which represents the relationship between the interface yield strength and the dislocation-accumulation length (estimated as 0.145 MPa·m^0.5^) [2], $\Delta G$ is the average shear modulus for the FCC and B2 phases, $\delta$ is a lattice-parameter-related parameter, which is obtained by the formula, $\delta=\Delta a/\bar{a}$, where $\bar{a}$ represents the average lattice parameter of FCC and B2 [13] phases. $\varepsilon$ represents the residual-elastic strain at the heterogeneous interface, which can be approximated as 0.76$\delta$ [2]. $\lambda$ is the grain dimension, which determines the misfit stresses, and is identified with the thickness of the FCC phase in the present alloys. $\Delta\gamma$ is the SFE difference between the FCC and B2 phases, based on the SFE ranges of FCC and BCC MPEAs obtained from atomic simulations by Rao et al. [14, 15]: The SFE range for the FCC lattice is 380 – 418 mJ·m^−2^ , and that for the BCC lattice is 20 – 40 mJ·m^−2^ . For ease of calculation in this study, the average SFEs for the FCC and BCC lattices were taken as 399 mJ·m^−2^ and 30 mJ·m^−2^, respectively. The phase-boundary strengthening contribution values of the vermicular and lamellar EMPEAs estimated by the above formulas are approximately ~ 365 MPa and ~ 197 MPa, respectively.

In summary, the theoretically estimated strength values of the vermicular and lamellar EMPEAs are 925 MPa and 650 MPa, respectively, which closely align with the experimental results. It is important to emphasize that due to minor differences in compositions and properties, the intrinsic-strength values of the vermicular and lamellar EMPEAs in the FCC phase are similar. However, concerning phase-boundary strengthening, the vermicular EMPEA exhibits a significantly enhanced effect compared with the lamellar EMPEA.

Supplementary Note 4. Stress distribution with PFM method.

In typical lamellar EMPEAs, the spatial distribution of the stress might be inhomogeneous due to the anisotropic nature of the lamella, while the vermicular microstructure can alleviate such spatial inhomogeneity. Besides, in dual-phase EMPEAs with an external strain, it can be expected that the strain in the high modulus phase would introduce higher stress according to the Hooke’s law. This inhomogeneous distribution of stress may not be unfavorable for the co-deformation of the phases, while the similar modules of the phases in our EMPEA can alleviate it. To demonstrate these, we simulated the stress distribution with the phase-field microelasticity (PFM) method on vermicular (AlCrFe_2_)_65_Ni_35_ EMPEA and lamellar AlCoCrFeNi_2.1_ EMPEA under an external strain that caused the same uniaxial tension $\sigma_{yy}^{appl}$ in y direction as shown in Figure S4, where the stresses are calculated by the linear Hooke’s law $\sigma_{ij}=C_{ijkl}\left(\varepsilon_{ij}-\varepsilon_{ij}^{*}\right)$. The lamellar EMPEA shows a strip-like region (denoted by the green arrow in Figure S4e) with abnormal stress distribution that the BCC phase bears less stress than BCC in other regions while the FCC phase bears more. It can be observed that the reason is that the longitudinal direction of the lamella is parallel to the direction of the external stress, and therefore, the soft FCC phase cannot transfer the stress to the hard BCC phase. Such regions could be the weak points and origin of cracks during deformation, and thus, the alloy would fail before the full potential of its strength and ductility is demonstrated. Meanwhile, no such region is observed in the vermicular EMPEA. Besides, the simulation results indicate a more homogeneous distribution of stress in the FCC and BCC phases in the vermicular EMPEA (Figure S4c) compared to that of lamellar EMPEA (Figure S4e). To quantitatively show this phenomenon, we calculated the ratio of average stress in BCC over that of FCC phase for different microstructures, $\sigma_{yy,ave}^{BCC}/\sigma_{yy,ave}^{FCC}$, and the result shows that the vermicular EMPEA significantly weakens the stress concentration (2.2186 for vermicular, 5.1347 for lamellar EMPEA). This could be attributed to closer elastic moduli of FCC and BCC phases as illustrated in Supplementary Note 5, as well as the increased phase boundary density, which facilitates the stress transfer. We expect that the more homogeneous distribution may contribute to the co-deformation of the FCC and BCC/B2 phases.

Supplementary Note 5. Calculation of elastic properties.

The elastic constants were calculated based on a density-functional theory, as implemented in the exact muffin-tin orbital coherent potential approximation (EMTO-CPA) code [16]. The EMTO method [17] is an improved and screened Korringa–Kohn–Rostoker method that calculates the one-electron Kohn–Sham states within the scalar relativistic approximation and the soft-core scheme. The total energy was calculated using the full charge density technique [18]. Electronic exchange–correlation was described within the generalized gradient approximation using the Perdew–Burke–Ernzerhof parametrization [19]. A 21 × 21 × 21 Monkhorst–Pack [20] k-point grid was used in the integrations over the Brillouin zone. A convergence criterion of 10^−6^ Ry was employed for both electronic and Fermi energy calculations. Substitutional disorder was treated using the coherent potential approximation (CPA) [21]. The limit to the number of self-consistent CPA iterations was set to 20, with a convergence tolerance of 10^−6^ Ry^−1^ for Green’s function elements. Here, due to the unknown and possibly changing exact order and composition of the FCC and BCC phases during the eutectic solidification, the modulus of the disordered FCC and BCC configuration with the composition of the bulk (AlCrFe_2_)_65_Ni_35_ EMPEA are calculated. The three independent components, $C_{11}$,$C_{12}$, and $C_{44}$, of the second-order elastic tensors for the BCC and FCC phases were computed using the strain-energy method. Three homogeneous deformation modes—one dilatation (volume scaling) and two volume-preserving (orthorhombic and monoclinic)—were applied to the fully relaxed unit cells of the crystals to compute the energy of the crystal at various strains for each deformation mode. The results of DFT calculation are validated against the experiment results in terms of lattice parameters with a good agreement, as shown in Table S5. The elastic constants were then obtained by fitting polynomials to the strain-energy values and computing the fitting coefficients and shown in Table S5. The direction dependence of Young’s moduli was carried out by a Python-based open-source module [22]. The 3-dimensional visualization and corresponding projections are shown in Figure S5, indicating a negligible modulus misfit for the (AlCrFe_2_)_65_Ni_35_ EMPEA and a considerable modulus misfit for the AlCoCrFeNi_2.1_ EMPEA despite the FCC and BCC shows different crystal lattice parameters. A possible reason is the complex dependencies of elastic properties on the alloying effect of aluminum [23]. Such anomalous characters may possibly lead to close elastic moduli for FCC and BCC phases, which is also reported by Vitos et al. in Al*_x_*CrMnFeCoNi (0 ≤ *x* ≤ 5) high entropy alloys and Zhai et al. in (CoCrNi)_100−_*_x_*Al*_x_* (0 ≤ *x* ≤ 28 at. %) high entropy alloys [24, 25].

Supplementary Note 6. EBSD crystallographic orientation analysis.

In lamellar EMPEA, the inverse pole figures (IPFs) are used to reveal the preferred orientations of the FCC and B2 phases in the lamellar EMPEA as shown in Figure S9(a-d). It is found that according to the IPFs of the Y- and Z-directions, the FCC and B2 phases maintain a Kurdjumov‒Sachs (K‒S) type orientation relationship, i.e. <111>FCC//<110>B2 and $\text{\{1}\bar{1}\text{0\}FCC}\text{//\{}\bar{1}\text{11\}B2}$. This result is consistent with the reported orientation relationship of the lamellar eutectic of similar alloy systems [26, 27]. This crystallographic "locking" effect is in agree with the phase-field simulation result and promotes the growth of the lamellar structure during solidification. Based on the IPFs of the vermicular EMPEA, it is found that the FCC and B2 phases do not meet any known orientation relationship, as shown in Figure S9(e-h). This absence of crystallographic locking allows the eutectic structure to grow in a non-directional manner, leading to the twisted vermicular morphology as revealed by the phase-field simulation.

Supplementary Note 7. Free-energy-density functional.

The free-energy-density functional reflects the thermodynamic interactions of liquid and eutectic phases, and is given by a double-well formalism:

(17)

where $\rho$, $a$, $b$, $\omega$, $\alpha$, and $\beta$ are dimensionless coefficients and $\Delta T$denotes the non-dimensional supercooling temperature. To fit the coefficients, thermodynamic quantities are related in terms of the mean-field theory [28]. The term is introduced to include the dendritic instabilities [29]. Under the equilibrium condition, the free energy coefficients by determining the minimum with , where we obtain the solutions for liquid and solid phases as:

|  |  | (18) |
| --- | --- | --- |
|  |  | (19) |

Additionally, the chemical potential is determined by:

|  |  | (20) |
| --- | --- | --- |

with $c_{1}$ and $c_{2}$ denoting the composition of eutectic phases, respectively. The$\mu_{0}$is given by:

|  | $\mu_{0}=\pm\frac{2\alpha b^{1/2}\left(\rho/a\right)^{1/2}}{\left[2\beta\left(\rho/a\right)^{1/2}-\omega\right]^{1/2}}\left(\Delta T-\Delta T_{E}\right)$ | (21) |
| --- | --- | --- |

where $\Delta T_{E}$ is the eutectic temperature and obtained by

|  | $\Delta T_{E}\approx\frac{\left[2\beta\left(\rho/a\right)^{1/2}-\omega\right]^{1/2}}{8\alpha b\left(\rho/a\right)^{1/2}}$ | (22) |
| --- | --- | --- |

Accordingly, the above parameters are fitted on the basis of dimensionless composition and thermal process for eutectic solidification, as listed in Table S4 [28]. Due to the lack of the thermodynamic data on the Al–Cr–Fe–Ni multi-component system, it was considered a pseudo-binary system of Al–TM (TM = transition metal). The interactions between Al and TM elements are significantly stronger than those among TM elements, and the latter can be neglected in simulations [30, 31] which justifies our simplification.

Supplementary Note 8. Numerical implementation of the phase-field model.

The PF model coupled with the PFM method was conducted through a two-dimensional finite-difference solver utilizing an explicit Euler integration scheme. The PFM solver calculated the elastic equilibrium in the computational field using an iterative algorithm based on FFT [32]. Here, all simulations were carried out on a square non-dimensional $256\times256$ grid with a uniform mesh size of $\Delta x=1.3$. Periodic-boundary conditions were applied to the computational field. The Langevin terms were presented in the form of Gaussian distributions with correlation functions, and$\left\langle\eta_{c}\left(\vec{r},t\right)\eta_{c}\left(0,0\right)\right\rangle=2Mk_{b}T\nabla^{2}\delta\left(\vec{r}\right)\delta\left(t\right)$ [33], where $k_{b}$ is the Boltzmann’s constant and $T$ is the temperature. $\delta\left(\vec{r}\right)$ and $\delta\left(t\right)$ denote the Kronecker delta functions with respect to coordinate and time, respectively. In the simulations, the Langevin noise was applied as a non-dimensional Gaussian random distribution with a magnitude of 0.29 to capture nucleation, with the time step set to $\Delta t=0.05$. The thermodynamic parameters that appeared in the PF model are non-dimensional and related to the eutectic nature of the system by a mean-field theory, based on the equation [28]. Here, $F_{meanfield}$ denotes the free-energy functional at the extrema of two field variables. The non-dimensional parameters for the liquid–solid mobility, $L$, and chemical mobility, $M$, were inserted into the governing equations with values of $L=1$, $M=0.4$. By introducing the characteristic time, $\tau'$, characteristic length scale, $l_{0}$, and mobility normalization factor, $M_{n}$, dimensionalization was performed as $t'=\tau't$, $M'=MM_{n}/V_{m}$, $L'=LM_{n}V_{m}/l_{0}^{2}$, and , where $V_{m}$ is the molar volume, and $RT$ is the gas constant times temperature equaled to 8.314. In our simulations, microstructural evolution was observed for a time duration of $t=1000$ timesteps through a MATLAB algorithm. The initial condition simulates a nuclei-free undercooled liquid state characterized by the field variables, and $c\left(\vec{r},0\right)=\xi\left(\vec{r}\right)$, where $\xi\left(\vec{r}\right)<\left|0.01\right|$ is a uniform random noise approximating the stochastic nature of the material. The equilibrium of the system’s elastic energy was subsequently solved for the case in which two solid phases grow competitively. In eutectic phases, elastic inhomogeneities were determined by field variables, with each phase identified with three independent components, $C_{11}$,$C_{12}$, and $C_{44}$, for the second-order elastic tensors. The calculations for these elastic constants were based on the density functional theory, as described in Supplementary Note 5. All the parameters used in the simulations are listed in Table S4 and Table S5.

Supplementary Figures:

**
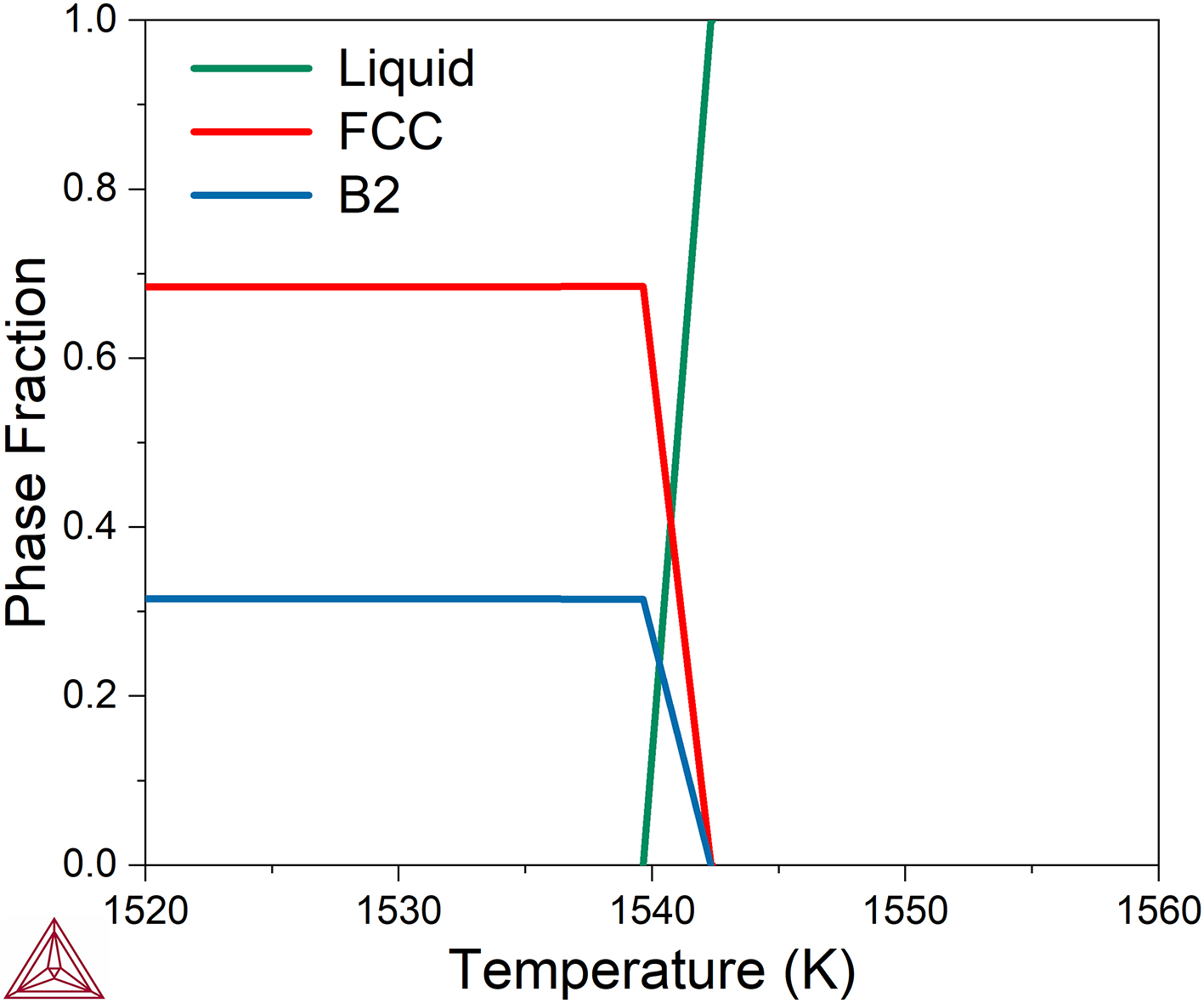
**

Figure S1. Vertical-section phase diagram near the melting point calculated using the CALPHAD method.


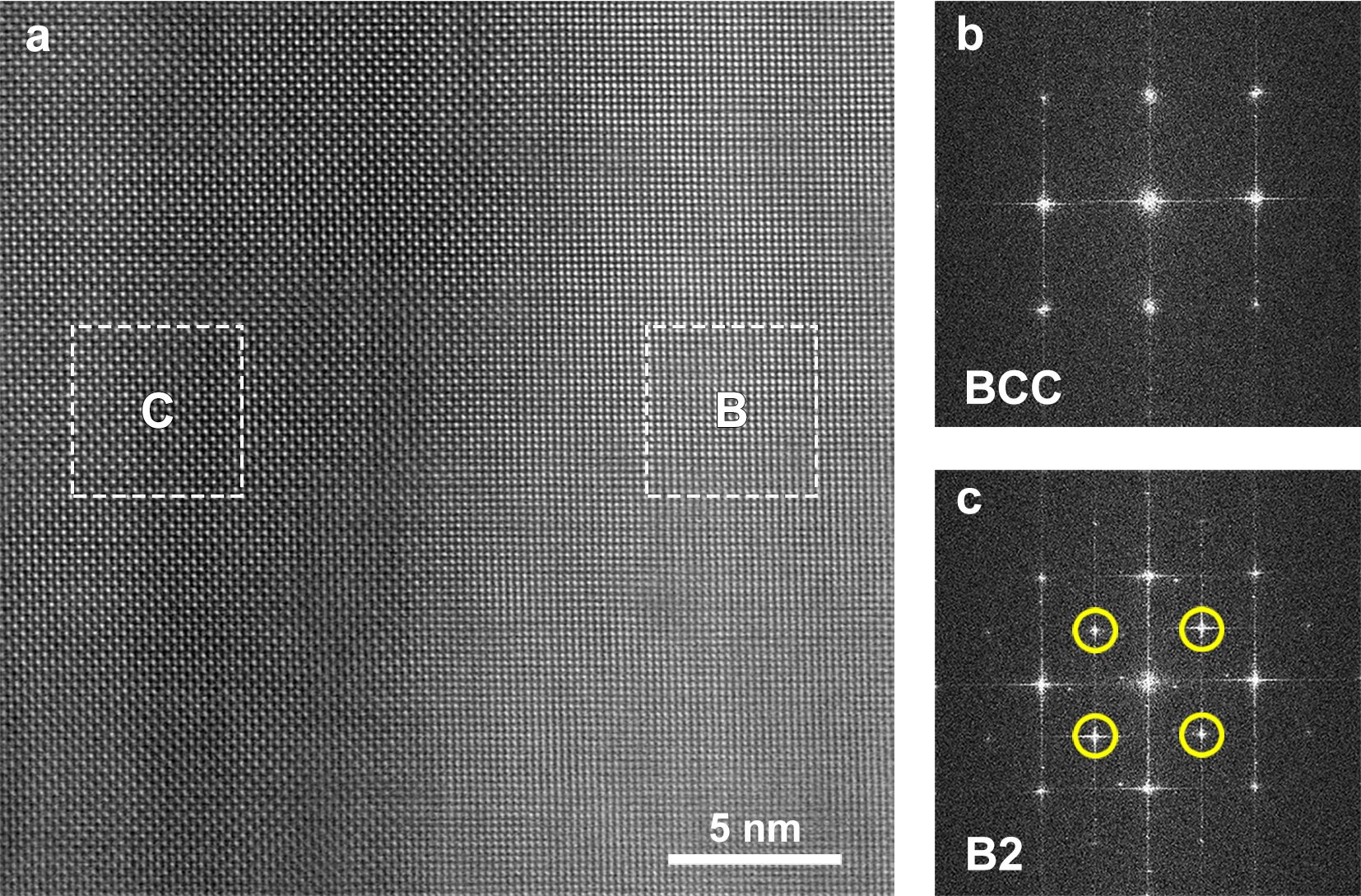


Figure S2. Atomic-scale image of the BCC–B2 phase boundary observed by aberration-corrected STEM-HAADF. (a): Atomic-scale aberration-corrected STEM-HAADF image of the boundary between the disordered BCC and ordered BCC phases along the [001]_BCC_ zone axis; (b and c): FFT image of the disordered BCC (area labeled ‘B’ in a) and ordered BCC phase (area labeled ‘C’ in a), where the orderedness is confirmed by the presence of the (100) pattern in c (indicated by yellow circles).


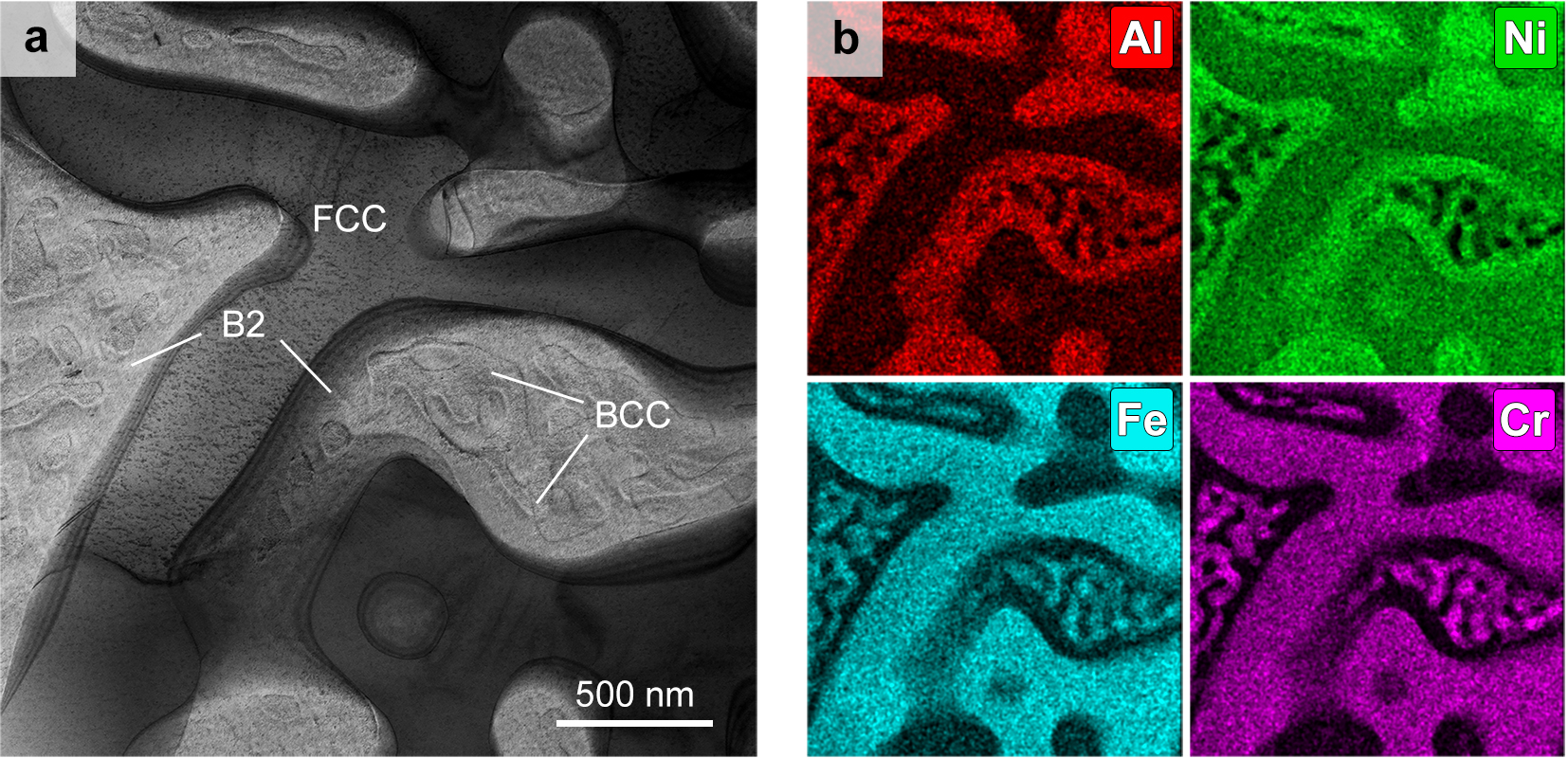


Figure S3. Phase structures and elemental distributions of vermicular EMPEA. (a): BF-STEM image of the vermicular structure; (b): STEM-EDS maps showing the elemental distribution of the phases in a. The results show that the FCC phase is enriched in Fe, Cr, and Ni, the BCC phase is enriched in Fe and Cr, and the B2 phase is enriched in Al and Ni.


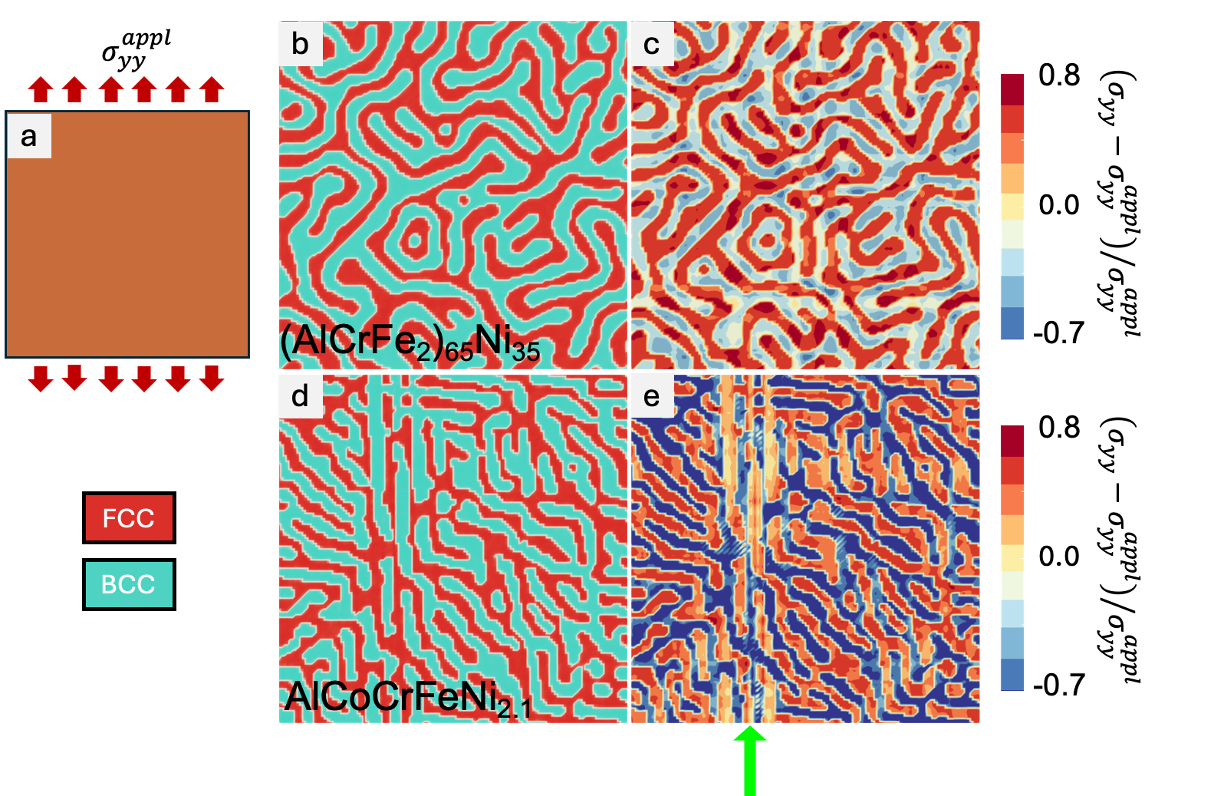


**Figure S4. Distribution of stress in vermicular and lamellar EMPEAs under uniaxial tension in y direction.** (a) Schematics of the system. (b) Microstructure of vermicular EMPEA. The corresponding calculated stress field $\left(\sigma_{yy}-\sigma_{yy}^{appl}\right)/\sigma_{yy}^{appl}$ is depicted in (c). (d) Microstructure of lamellar EMPEA. The corresponding computational stress field $\left(\sigma_{yy}-\sigma_{yy}^{appl}\right)/\sigma_{yy}^{appl}$ is depicted in (e). The stripe-like region with abnormal stress distribution is indicated by the green arrow.


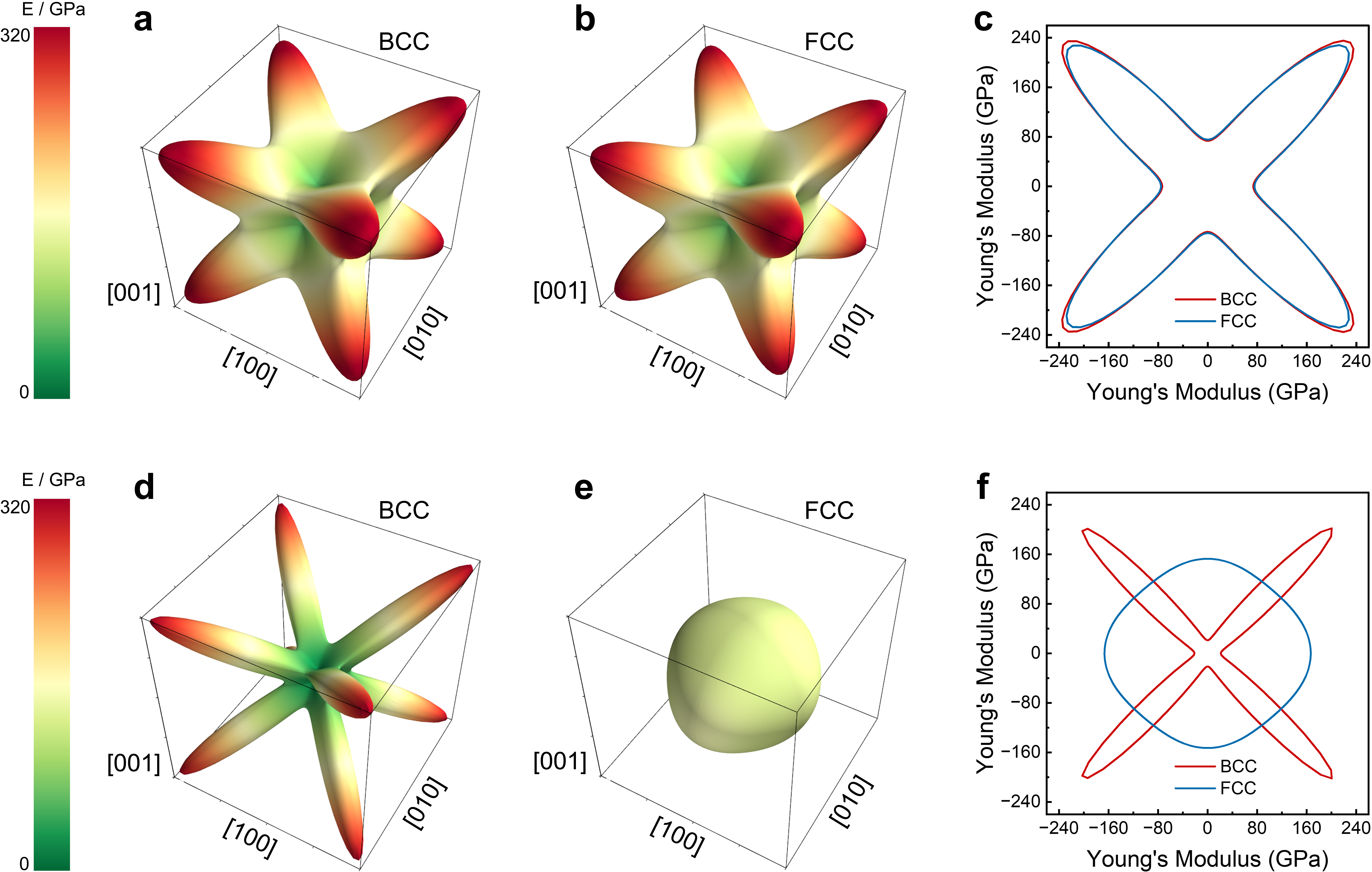


Figure S5. 3D representation of directional dependence of Young’s modulus for FCC and BCC phases in EMEPAs. (a and b): vermicular EMPEA, and (d and e): lamellar EMPEA; (c and f): 2D projections of the direction-dependent Young’s modulus with respect to the (001) direction for vermicular and lamellar EMEPAs, respectively.


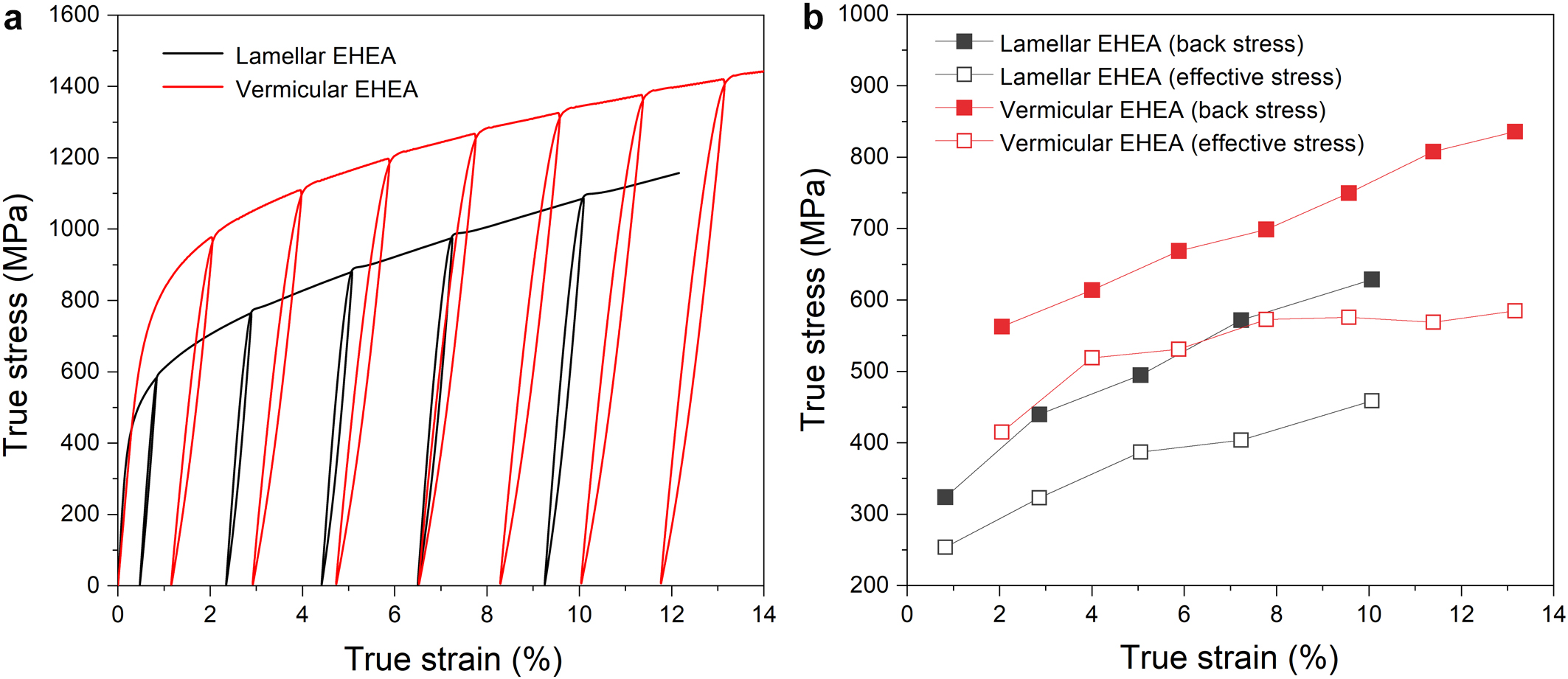


Figure S6. Back-stress analysis of the lamellar and vermicular EMPEAs. (a): Loading–unloading–reloading true stress–strain curves of the lamellar and vermicular EMPEAs. (b): Variation of the back-stress and effective stress with the true strain in the lamellar and vermicular EMPEAs. Compared with the lamellar EMPEA, the vermicular EMPEA shows a more significant Bauschinger effect.


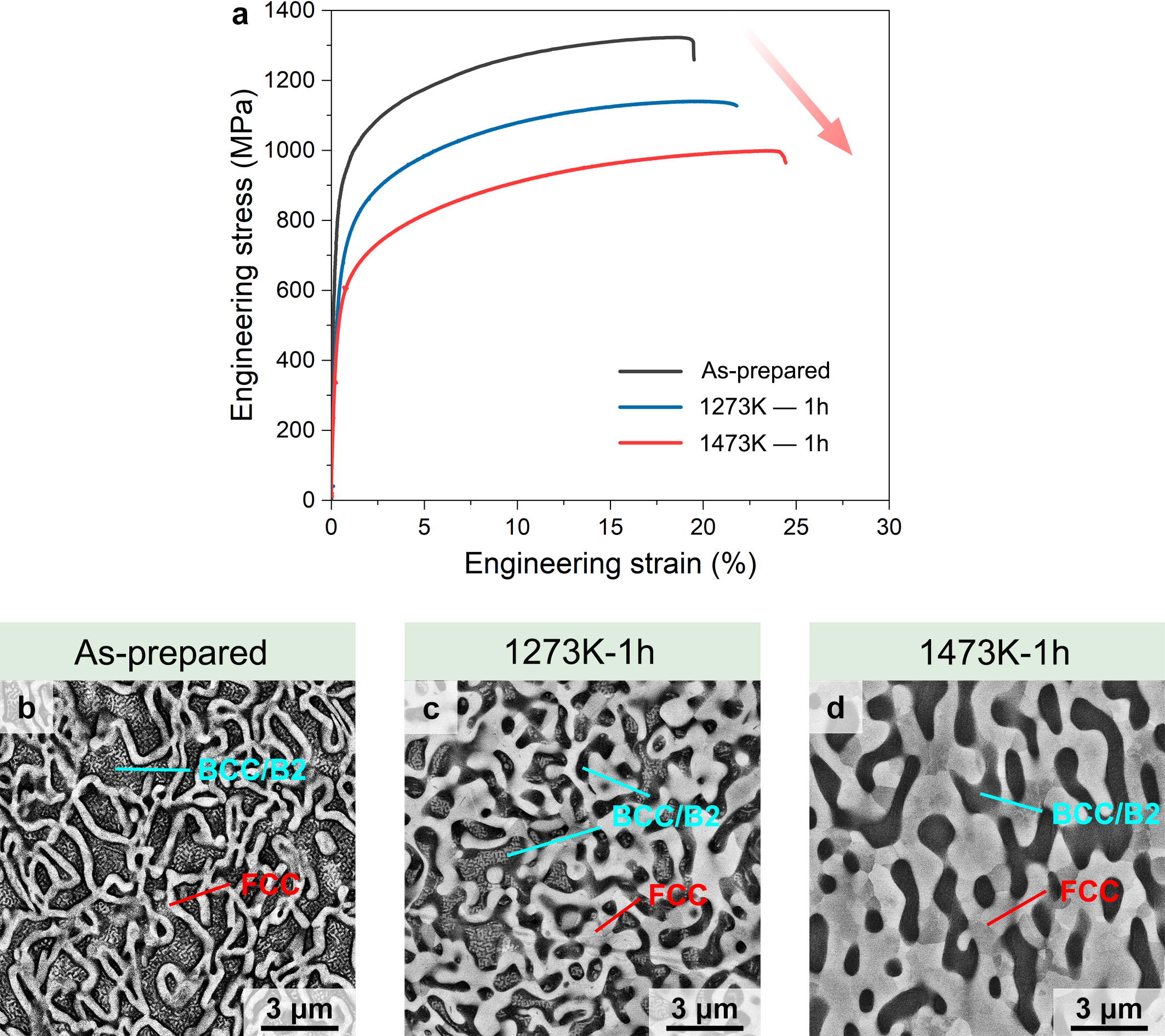


Figure S7. Engineering stress–strain curves and phase structure of a vermicular EMPEA before and after solution treatment. (a): Room-temperature engineering stress-strain curves of the vermicular EMPEA after a solid-solution treatment at 1,273 K and 1,473 K (with stress-strain curves before the heat treatment for comparison); (b): SEM image of the initial structure of the vermicular EMPEA; (c and d): SEM images of the vermicular EMPEA after 1-hour solid-solution treatments at 1,273 K and 1,473 K, respectively.


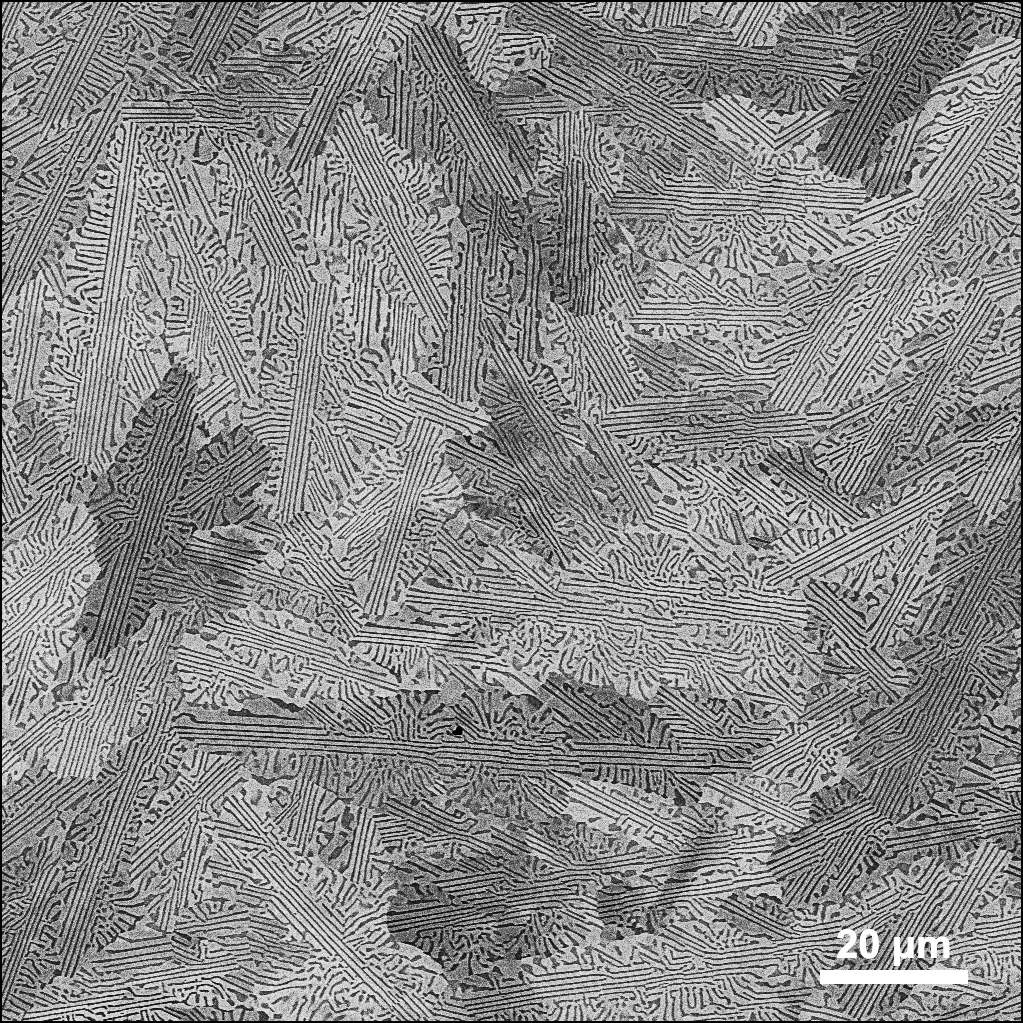


Figure S8. SEM image of a typical lamellar EMPEA. Using the same preparation method as for the vermicular EMPEA, the AlCoCrFeNi_2.1_ (at.%) lamellar EMPEA was repeatedly vacuum-arc melted five times to achieve homogenization. Subsequently, a rectangular ingot with dimensions of 70 mm × 10 mm × 10 mm was prepared by water-cooled copper-mold suction casting.


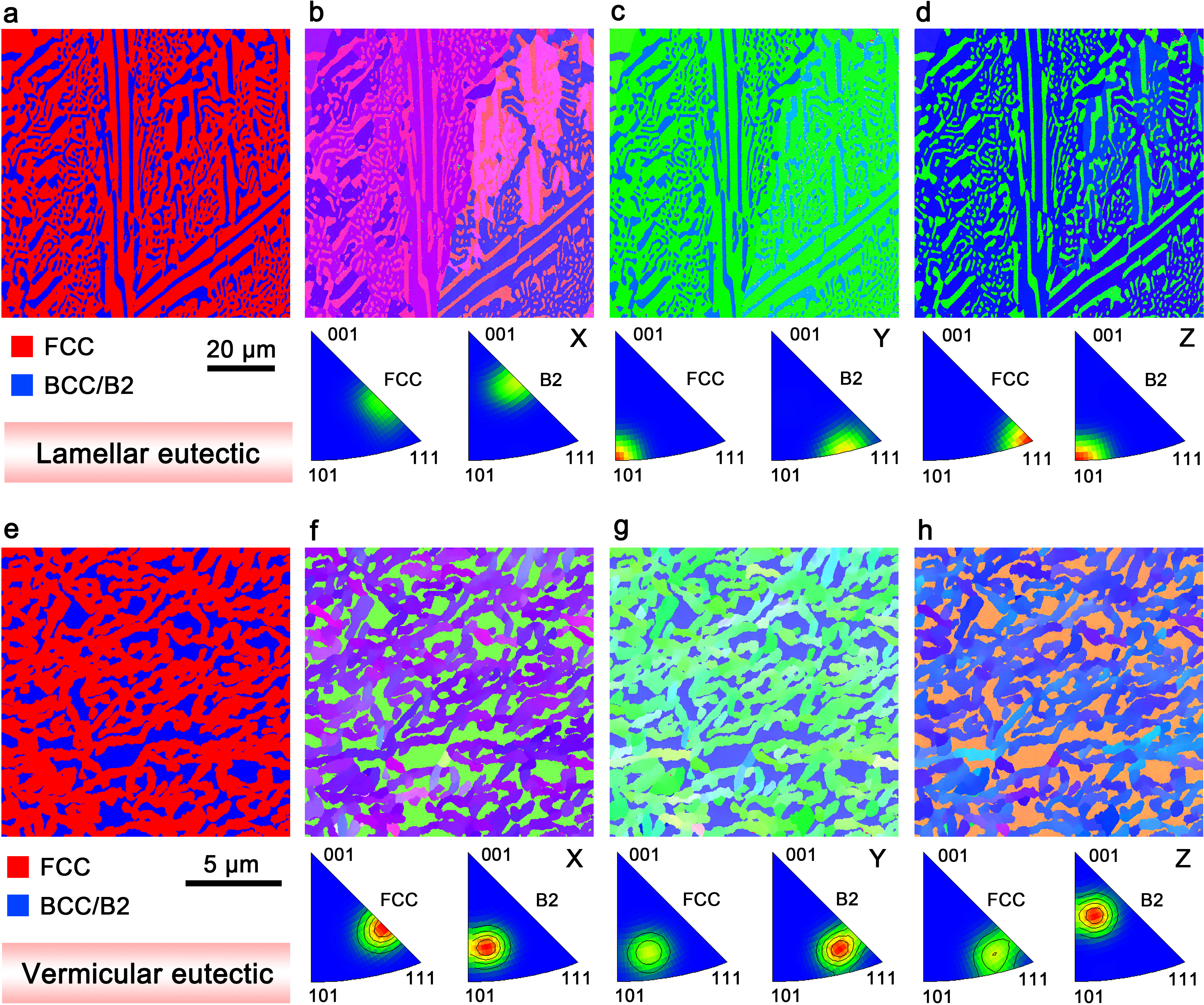


Figure S9. EBSD analysis of lamellar and vermicular EMPEAs. (a): Phase map of the lamellar EMPEA; (b-d): IPF figures of the lamellar eutectic along the X, Y, and Z directions, respectively; (e): Phase map of the vermicular EMPEA; (f-h): IPF figures of the vermicular eutectic along the X, Y, and Z directions, respectively.


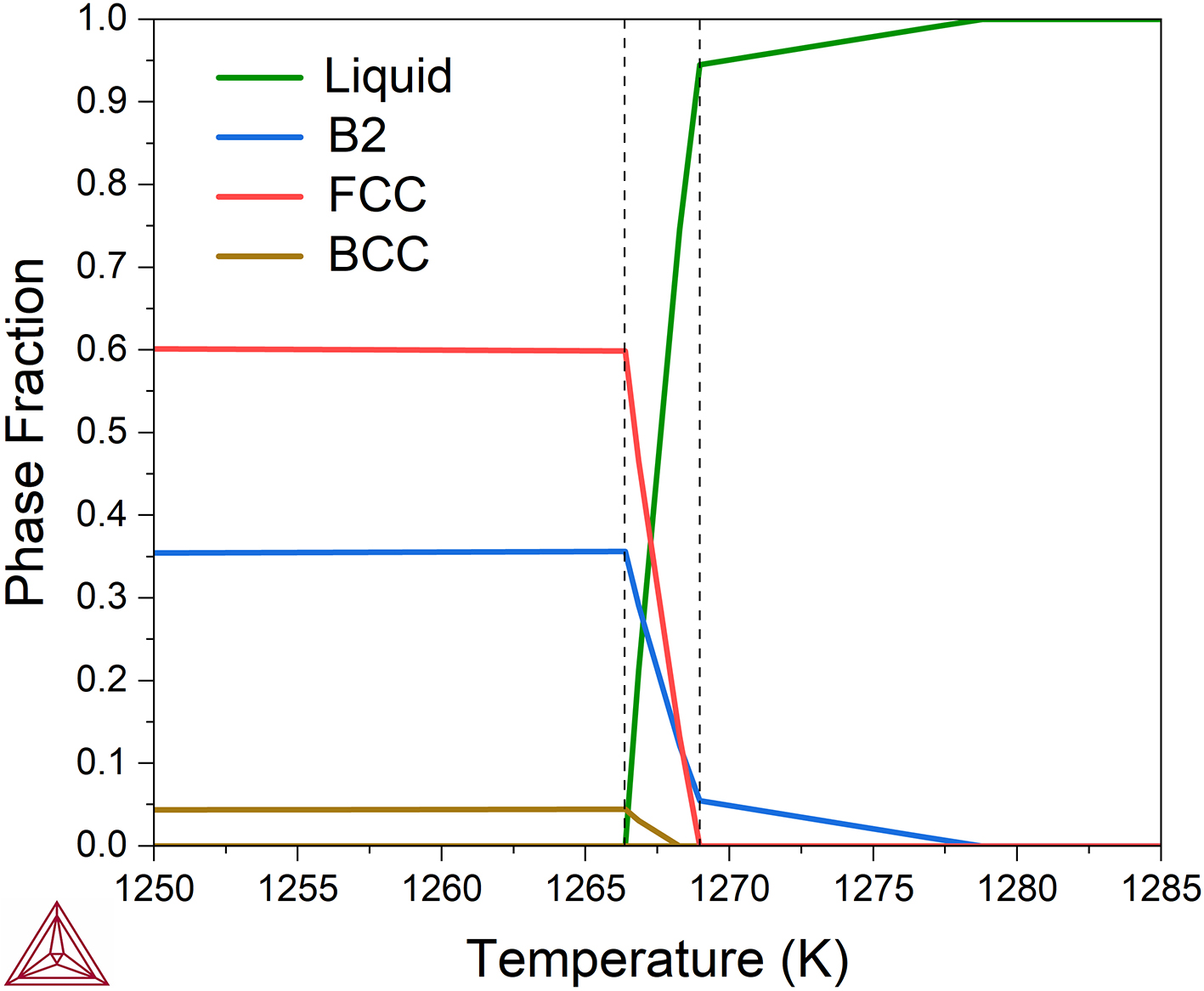


Figure S10. Vertical-section phase diagram calculated on the AlCrFe_2_Ni_2_ alloy using the CALPHAD method.


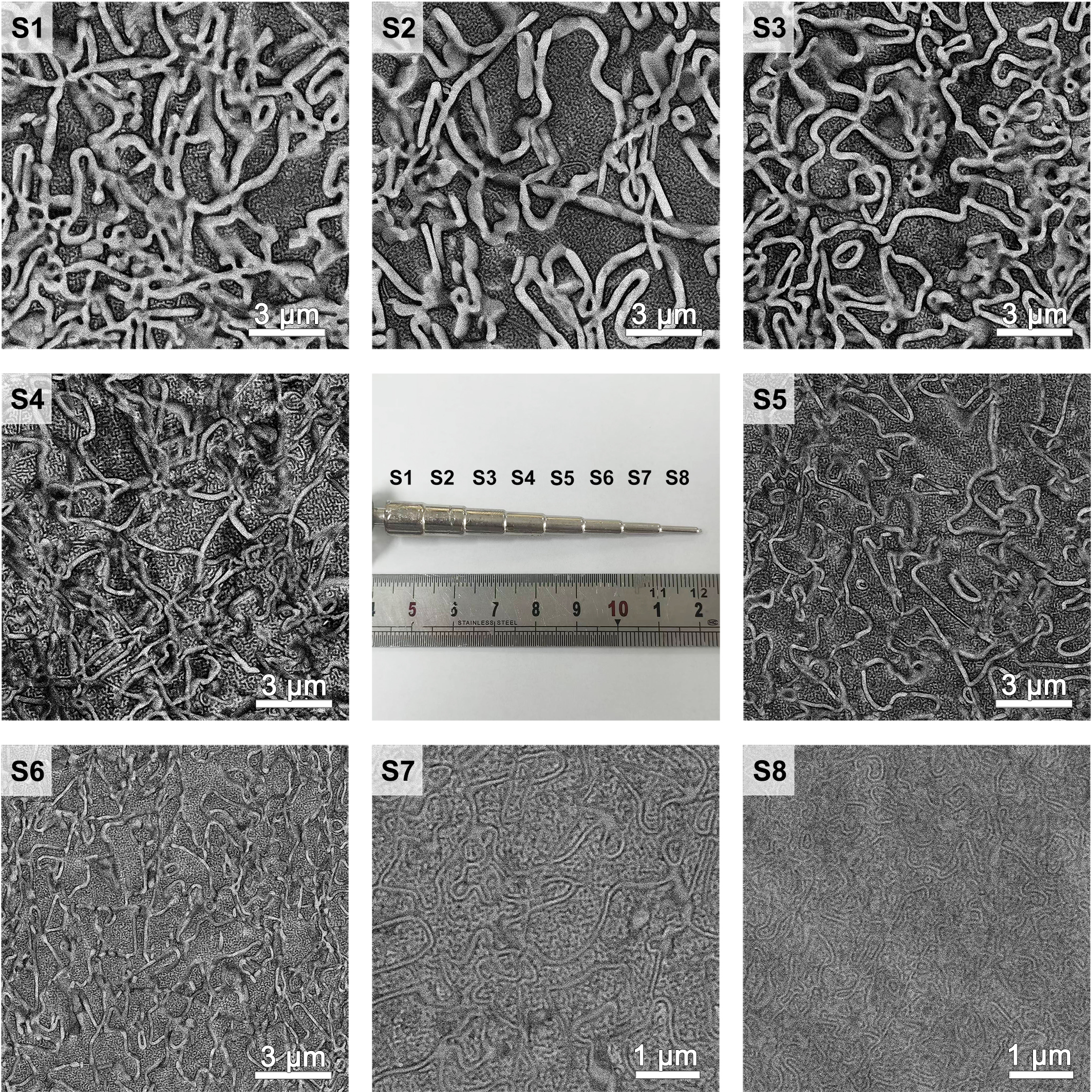


Figure S11. Vermicular microstructures observed under different cylindrical sample diameters.


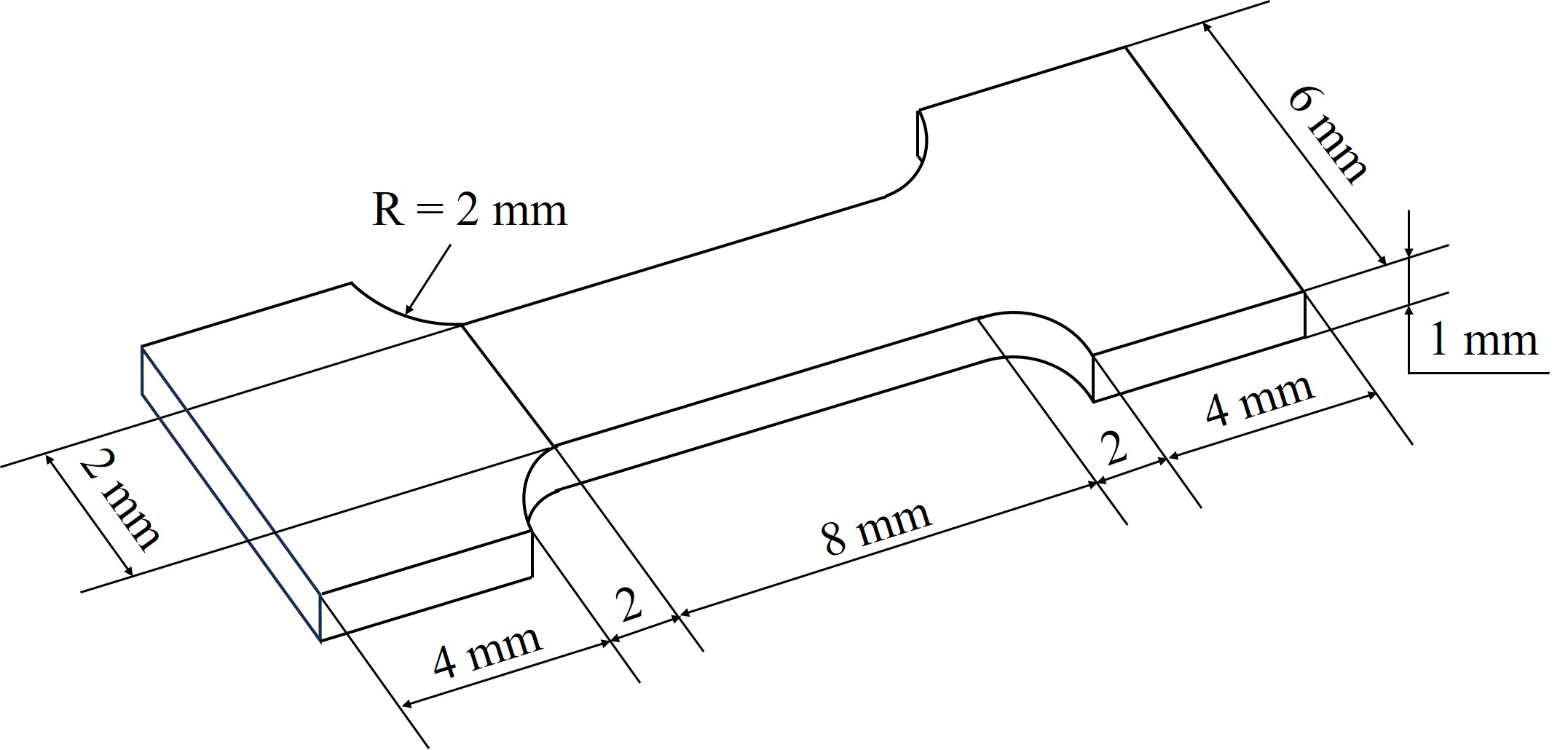


Figure S12. Detailed geometry of a dog-bone-shaped sample.

Supplementary Tables:

Table S1. Composition of phases at 1,250 K calculated by the CALPHAD method.

| Phase | Al fraction (at.%) | Cr fraction (at.%) | Fe fraction (at.%) | Ni fraction (at.%) |
| --- | --- | --- | --- | --- |
| FCC | 7.38 | 22.40 | 41.18 | 29.03 |
| B2 | 32.78 | 3.91 | 16.51 | 46.80 |
| BCC | 4.03 | 46.76 | 39.76 | 9.45 |

**Table S2.** **Detailed tensile properties of the vermicular EMPEA and Al-Co-Cr-Fe-Ni system lamellar EMPEAs.**

| EMPEA | Yield strength (MPa) | Tensile strength (MPa) | Elongation (%) | Refs. |
| --- | --- | --- | --- | --- |
| (AlCrFe_2_)_65_Ni_35_ | 920 ± 25 | 1,322 ± 50 | 19.5 ± 2 | - |
| AlCoCrFeNi_2.1_ | ~ 512 | ~ 1,080 | ~ 16.2 | [34] |
| AlCoCrFeNi_2.1_ | ~ 546 | ~ 1,046 | ~ 17.7 | [35] |
| Al_17_Co_14.3_Cr_14.3_Fe_14.3_Ni_40.1_ | ~ 479 | ~ 1,067 | ~ 14.0 | [36] |
| Al_17_Co_28.6_Cr_14.3_Fe_14.3_Ni_25.8_ | ~ 473 | ~ 1,001 | ~ 14.8 | [36] |
| Al_18_Co_30_Cr_11_Fe_11_Ni_30_ | ~ 615 | ~ 1,000 | ~ 16.5 | [37] |
| Al_1.25_CoCrFeNi_3_ | ~ 535 | ~ 1,061 | ~ 17.2 | [38] |
| Al_20_Co_36_Cr_4_Fe_4_Ni_36_ | ~ 483 | ~ 1,005 | ~ 8.8 | [39] |
| Al_17_Cr_8_Fe_32_Ni_43_ | ~ 490 | ~ 951 | ~ 15.2 | [40] |
| Al_16.6_Cr_12_Fe_28_Ni_43.4_ | ~ 549 | ~ 979 | ~ 12.1 | [40] |
| Al_16.4_Cr_16_Fe_24_Ni_43.6_ | ~ 575 | ~ 1,012 | ~ 12.6 | [40] |
| Al_16_Cr_20_Fe_20_Ni_44_ | ~ 654 | ~ 1,050 | ~ 10.8 | [40] |
| AlCrFeNi_3_ | ~ 626 | ~ 1,200 | ~ 10.1 | [41] |
| Al_0.8_CrFeNi_2.2_ | ~ 479 | ~ 956 | ~ 12.7 | [42] |
| AlCrFe_2_Ni_2_ | ~ 780 | ~ 1,228 | ~ 15 | [43] |
| Al_19_Co_20_Fe_20_Ni_41_ | ~ 577 | ~ 1,103 | ~ 18.7 | [44] |
| Al_19_Co_20_Fe_20_Ni_41_ | ~ 520 | ~ 1,050 | ~ 16.0 | [45] |
| Al_19.25_Co_18.86_Fe_18.36_Ni_43.53_ | ~ 486 | ~ 956 | ~ 10 | [46] |
| Al_0.9_CoFeNi_2_ | ~ 500 | ~ 1,005 | ~ 6.2 | [47] |
| Al_20.33_Co_21.60_Fe_30.45_Ni_27.62_ | ~ 632 | ~ 1,022 | ~ 13.2 | [48] |
| Al_0.9_CoFeNi_2.1_ | ~ 645 | ~ 1,033 | ~ 6.9 | [49] |
| Al_18_Co_24_Cr_20_Ni_38_ | ~ 543 | ~ 1,005 | ~ 8.1 | [50] |
| Al_17.5_Co_24_Cr_20_Ni_38.5_ | ~ 530 | ~ 1,028 | ~ 14.5 | [50] |
| Al_19.3_Co_15_Cr_15_Ni_50.7_ | ~ 699 | ~ 1,127 | ~ 10.3 | [51] |
| Al_17.5_Co_20_Cr_20_Ni_42.5_ | ~ 741 | ~ 1,272 | ~ 14.4 | [51] |
| Al_16.3_Co_25_Cr_25_Ni_33.7_ | ~ 655 | ~ 1,109 | ~ 13.6 | [51] |
| Al_17_Cr_17_Co_33_Ni_33_ | ~ 559 | ~ 1,028 | ~ 19.1 | [52] |

Table S3. Thermophysical parameters of the (AlCrFe_2_)_65_Ni_35_ EMPEA. A multiphase microstructure was predicted from the value of $\Delta H_{mix}-\delta$ [53, 54], and the FCC + BCC multiphase microstructure was predicted from the valence electron concentration [55].

| Parameter | Equation | Calculated value | Ref. |
| --- | --- | --- | --- |
| Mixing entropy | $\Delta S_{mix}=-R\sum_{i=1}^{n} c_{i}\ln c_{i}$ | 11.0016  J K^−1^ mol^−1^ | [56] |
| Mixing enthalpy | $\Delta H_{mix}=4\sum_{i,j=1}^{i,j=n} \Delta H_{ij}^{mix}c_{i}c_{j}$ | − 11.0988  kJ mol^−1^ | [56] |
| Valence electron concentration | $VEC=\sum_{i=1}^{n} c_{i}VEC_{i}$ | 7.5625 | [57] |
| Atomic size mismatch | $\delta=\sqrt{\sum_{i=1}^{n} c_{i}\left(1-r_{i}/\bar{r}\right)^{2}}$ | 5.4252% | [58] |

The individual terms are defined as follows: *C_i_*(*C_j_*) = atomic percentage of the *i*‑th (*j*‑th) component; *r_i_* = atomic radius; and (*VEC*)*_i_* = valence electron concentration.

Table S4. Parameters used in phase-field simulations.

| Parameter type | Parameter | Value |
| --- | --- | --- |
| PF model | $L$ | 1.0 |
|  | $M$ | 0.4 |
|  |  | 0.125 |
|  | $\kappa_{c}$ | 0.5 |
|  | $\rho$ | 1.0 |
|  | $a$ | 1.0 |
|  | $b$ | 1.0 |
|  | $\omega$ | 0.0 |
|  | $\alpha$ | 1.0 |
|  | $\beta$ | 1.0 |
| PFM model | $\varepsilon^{0}$ | 0.223 |

Table S5. Lattice constants and second-order elastic tensors.

|  | FCC | BCC |
| --- | --- | --- |
| Lattice parameter by experiment | 3.598 Å | 2.876 Å |
| Lattice parameter by DFT | 3.593996 Å | 2.857628 Å |
| Relative difference | 0.11 % | 0.6 % |
| $C_{11}$ | 196.6 GPa | 201.4 GPa |
| $C_{12}$ | 154.3 GPa | 160.4 GPa |
| $C_{44}$ | 144.4 GPa | 149.3 GPa |

**References**

[1] D. Hull, D.J. Bacon, Introduction to Dislocations (Fifth Edition), Butterworth-Heinemann, Oxford, 2011, pp. 205-249.

[2] I. Basu, V. Ocelík, J.T. De Hosson, BCC-FCC interfacial effects on plasticity and strengthening mechanisms in high entropy alloys, Acta Materialia 157 (2018) 83-95.

[3] L. Li, Q. Fang, J. Li, B. Liu, Y. Liu, P.K. Liaw, Lattice-distortion dependent yield strength in high entropy alloys, Materials Science and Engineering: A 784 (2020) 139323.

[4] W. Li, D. Xie, D. Li, Y. Zhang, Y. Gao, P.K. Liaw, Mechanical behavior of high-entropy alloys, Progress in Materials Science 118 (2021) 100777.

[5] J.D. Eshelby, F.C. Frank, F.R.N. Nabarro, XLI. The equilibrium of linear arrays of dislocations, The London, Edinburgh, and Dublin Philosophical Magazine and Journal of Science 42(327) (1951) 351-364.

[6] R. Labusch, A Statistical Theory of Solid Solution Hardening, physica status solidi (b) 41(2) (1970) 659-669.

[7] I. Toda-Caraballo, P.E.J. Rivera-Díaz-del-Castillo, Modelling solid solution hardening in high entropy alloys, Acta Materialia 85 (2015) 14-23.

[8] R.L. Fleischer, Substitutional solution hardening, Acta Metallurgica 11(3) (1963) 203-209.

[9] X. Yang, L. Feng, X. Wang, R. Chen, G. Qin, Y. Su, Dual enhancement in strength and ductility of Al1.25CoCrFeNi3 eutectic high entropy alloy by directional solidification, Materials Characterization 214 (2024) 114122.

[10] T. Xiong, S. Zheng, J. Pang, X. Ma, High-strength and high-ductility AlCoCrFeNi2.1 eutectic high-entropy alloy achieved via precipitation strengthening in a heterogeneous structure, Scripta Materialia 186 (2020) 336-340.

[11] A. Misra, J.P. Hirth, R.G. Hoagland, Length-scale-dependent deformation mechanisms in incoherent metallic multilayered composites, Acta Materialia 53(18) (2005) 4817-4824.

[12] S.I. Rao, P.M. Hazzledine, Atomistic simulations of dislocation–interface interactions in the Cu-Ni multilayer system, Philosophical Magazine A 80(9) (2000) 2011-2040.

[13] Y.P. Li, G.P. Zhang, W. Wang, J. Tan, S.J. Zhu, On interface strengthening ability in metallic multilayers, Scripta Materialia 57(2) (2007) 117-120.

[14] S.I. Rao, C. Varvenne, C. Woodward, T.A. Parthasarathy, D. Miracle, O.N. Senkov, W.A. Curtin, Atomistic simulations of dislocations in a model BCC multicomponent concentrated solid solution alloy, Acta Materialia 125 (2017) 311-320.

[15] S.I. Rao, C. Woodward, T.A. Parthasarathy, O. Senkov, Atomistic simulations of dislocation behavior in a model FCC multicomponent concentrated solid solution alloy, Acta Materialia 134 (2017) 188-194.

[16] L. Vitos, Computational quantum mechanics for materials engineers: the EMTO method and applications, Springer Science \& Business Media2007.

[17] L. Vitos, Total-energy method based on the exact muffin-tin orbitals theory, Physical Review B 64(1) (2001) 014107.

[18] L. Vitos, J. Kollr, H.L. Skriver, Full charge-density scheme with a kinetic-energy correction: Application to ground-state properties of the 4d metals, Physical Review B 55(20) (1997) 13521.

[19] J.P. Perdew, K. Burke, M. Ernzerhof, Generalized gradient approximation made simple, Physical review letters 77(18) (1996) 3865.

[20] H.J. Monkhorst, J.D. Pack, Special points for Brillouin-zone integrations, Physical review B 13(12) (1976) 5188.

[21] B.L. Gyorffy, Coherent-potential approximation for a nonoverlapping-muffin-tin-potential model of random substitutional alloys, Physical Review B 5(6) (1972) 2382.

[22] R. Gaillac, P. Pullumbi, F.X. Coudert, ELATE: an open-source online application for analysis and visualization of elastic tensors, J Phys Condens Matter 28(27) (2016) 275201.

[23] M.P. Agustianingrum, S. Yoshida, N. Tsuji, N. Park, Effect of aluminum addition on solid solution strengthening in CoCrNi medium-entropy alloy, Journal of Alloys and Compounds 781 (2019) 866-872.

[24] H. Zhang, X. Sun, S. Lu, Z. Dong, X. Ding, Y. Wang, L. Vitos, Elastic properties of AlxCrMnFeCoNi (0 ≤ x ≤ 5) high-entropy alloys from ab initio theory, Acta Materialia 155 (2018) 12-22.

[25] L. Li, S. Zhai, First-principles calculation of phase transitions and mechanical properties of (CoCrNi)100−xAlx (0 ≤ x ≤ 28 at. %) high-entropy alloys, AIP Advances 14(4) (2024) 045020.

[26] Q. Wang, Y. Lu, Q. Yu, Z. Zhang, The Exceptional Strong Face-centered Cubic Phase and Semi-coherent Phase Boundary in a Eutectic Dual-phase High Entropy Alloy AlCoCrFeNi, Scientific reports 8(1) (2018) 14910.

[27] X. Gao, Y. Lu, B. Zhang, N. Liang, G. Wu, G. Sha, J. Liu, Y. Zhao, Microstructural origins of high strength and high ductility in an AlCoCrFeNi2.1 eutectic high-entropy alloy, Acta Materialia 141 (2017) 59-66.

[28] F. Drolet, K.R. Elder, M. Grant, J.M. Kosterlitz, Phase-field modeling of eutectic growth, Phys Rev E Stat Phys Plasmas Fluids Relat Interdiscip Topics 61(6 Pt B) (2000) 6705-20.

[29] R. Kobayashi, Modeling and numerical simulations of dendritic crystal growth, Physica D: Nonlinear Phenomena 63(3) (1993) 410-423.

[30] J.L. Li, Z. Li, Q. Wang, C. Dong, P.K. Liaw, Phase-field simulation of coherent BCC/B2 microstructures in high entropy alloys, Acta Materialia 197 (2020) 10-19.

[31] S. Kolling, D. Gross, Simulation of microstructural evolution in materials with misfitting precipitates, Probabilistic Engineering Mechanics 16(4) (2001) 313-322.

[32] Y. Shen, Y. Li, Z. Li, H. Wan, P. Nie, An improvement on the three-dimensional phase-field microelasticity theory for elastically and structurally inhomogeneous solids, Scripta Materialia 60(10) (2009) 901-904.

[33] R. Zwanzig, Nonequilibrium Statistical Mechanics2001, pp. 3-29.

[34] P. Shi, W. Ren, T. Zheng, Z. Ren, X. Hou, J. Peng, P. Hu, Y. Gao, Y. Zhong, P.K. Liaw, Enhanced strength–ductility synergy in ultrafine-grained eutectic high-entropy alloys by inheriting microstructural lamellae, Nature Communications 10(1) (2019) 489.

[35] Y. Lu, X. Gao, L. Jiang, Z. Chen, T. Wang, J. Jie, H. Kang, Y. Zhang, S. Guo, H. Ruan, Y. Zhao, Z. Cao, T. Li, Directly cast bulk eutectic and near-eutectic high entropy alloys with balanced strength and ductility in a wide temperature range, Acta Materialia 124 (2017) 143-150.

[36] X. Jin, Y. Zhou, L. Zhang, X. Du, B. Li, A new pseudo binary strategy to design eutectic high entropy alloys using mixing enthalpy and valence electron concentration, Materials & Design 143 (2018) 49-55.

[37] D.H. Chung, J. Lee, Q.F. He, Y.K. Kim, K.R. Lim, H.S. Kim, Y. Yang, Y.S. Na, Hetero-deformation promoted strengthening and toughening in BCC rich eutectic and near eutectic high entropy alloys, Journal of Materials Science & Technology 146 (2023) 1-9.

[38] X. Yang, L. Feng, T. Liu, R. Chen, G. Qin, S. Wu, Tensile properties and strengthening mechanisms of eutectic high-entropy alloys induced by heterostructure, Materials Characterization 208 (2024) 113464.

[39] W. Jiao, T. Li, G. Yin, T. He, T. Li, Y. Lu, Hot deformation characteristics and microstructure evolution of Al20Co36Cr4Fe4Ni36 eutectic high entropy alloy, Materials Characterization 204 (2023) 113180.

[40] Z. Mao, X. Jin, Z. Xue, M. Zhang, J. Qiao, Understanding the yield strength difference in dual-phase eutectic high-entropy alloys, Materials Science and Engineering: A 867 (2023) 144725.

[41] Y. Dong, Z. Yao, X. Huang, F. Du, C. Li, A. Chen, F. Wu, Y. Cheng, Z. Zhang, Microstructure and mechanical properties of AlCoxCrFeNi3-x eutectic high-entropy-alloy system, Journal of Alloys and Compounds 823 (2020) 153886.

[42] X. Jin, J. Bi, L. Zhang, Y. Zhou, X. Du, Y. Liang, B. Li, A new CrFeNi2Al eutectic high entropy alloy system with excellent mechanical properties, Journal of Alloys and Compounds 770 (2019) 655-661.

[43] Y. Dong, X. Gao, Y. Lu, T. Wang, T. Li, A multi-component AlCrFe2Ni2 alloy with excellent mechanical properties, Materials Letters 169 (2016) 62-64.

[44] X. Jin, Y. Zhou, L. Zhang, X. Du, B. Li, A novel Fe20Co20Ni41Al19 eutectic high entropy alloy with excellent tensile properties, Materials Letters 216 (2018) 144-146.

[45] P. Shi, R. Li, Y. Li, Y. Wen, Y. Zhong, W. Ren, Z. Shen, T. Zheng, J. Peng, X. Liang, P. Hu, N. Min, Y. Zhang, Y. Ren, K. Liaw Peter, D. Raabe, Y.-D. Wang, Hierarchical crack buffering triples ductility in eutectic herringbone high-entropy alloys, Science 373(6557) (2021) 912-918.

[46] P. Shi, Y. Li, Y. Wen, Y. Li, Y. Wang, W. Ren, T. Zheng, Y. Guo, L. Hou, Z. Shen, Y. Jiang, J. Peng, P. Hu, N. Liang, Q. Liu, P.K. Liaw, Y. Zhong, A precipitate-free AlCoFeNi eutectic high-entropy alloy with strong strain hardening, Journal of Materials Science & Technology 89 (2021) 88-96.

[47] H. Jiang, D. Qiao, W. Jiao, K. Han, L. Yiping, P.K. Liaw, Tensile deformation behavior and mechanical properties of a bulk cast Al0.9CoFeNi2 eutectic high-entropy alloy, Journal of Materials Science & Technology 61 (2021) 119-124.

[48] Y. Huang, X. Chen, S. Ma, M. Wen, Y. Wang, Y. Wang, Double-wire arc additive manufacturing of AlCoFeNi eutectic high entropy alloy with balanced strength and plasticity, Materials Letters 363 (2024) 136312.

[49] X. Jin, Y. Liang, J. Bi, B. Li, Enhanced strength and ductility of Al0.9CoCrNi2.1 eutectic high entropy alloy by thermomechanical processing, Materialia 10 (2020) 100639.

[50] L. Ma, J. Wang, Z. Lai, Z. Wu, B. Yang, P. Zhao, Microstructure and mechanical property of Al56-xCo24Cr20Nix eutectic high-entropy alloys with an ordered FCC/BCT phase structure, Journal of Alloys and Compounds 936 (2023) 168194.

[51] Q. Liu, X. Liu, X. Fan, R. Li, X. Tong, P. Yu, G. Li, Designing novel AlCoCrNi eutectic high entropy alloys, Journal of Alloys and Compounds 904 (2022) 163775.

[52] C. Su, S. Konovalov, X. Chen, Y. Wang, Y. Jin, Microstructure and mechanical property of the novel Al17Cr17Co33Ni33 eutectic medium entropy alloy fabricated by powder plasma arc additive manufacturing, Materials Letters 361 (2024) 136074.

[53] Y.F. Ye, Q. Wang, J. Lu, C.T. Liu, Y. Yang, High-entropy alloy: challenges and prospects, Materials Today 19(6) (2016) 349-362.

[54] Q. He, Y. Yang, On Lattice Distortion in High Entropy Alloys, Frontiers in Materials 5 (2018).

[55] S. Guo, C. Ng, J. Lu, C.T. Liu, Effect of valence electron concentration on stability of fcc or bcc phase in high entropy alloys, J Appl Phys 109 (2011) 103505.

[56] S. Guo, C.T. Liu, Phase stability in high entropy alloys: Formation of solid-solution phase or amorphous phase, Prog. Nat. Sci. Mater. Int. 21(6) (2011) 433-446.

[57] S. Guo, C. Ng, J. Lu, C.T. Liu, Effect of valence electron concentration on stability of fcc or bcc phase in high entropy alloys, J. Appl. Phys. 109(10) (2011) 103505.

[58] X. Yang, Y. Zhang, Prediction of high-entropy stabilized solid-solution in multi-component alloys, Materials Chemistry and Physics 132(2-3) (2012) 233-238.
